# Excretion of Avenanthramides, Phenolic Acids and their Major Metabolites Following Intake of Oat Bran

**DOI:** 10.1002/mnfr.201700499

**Published:** 2017-12-29

**Authors:** Manuel Y. Schär, Giulia Corona, Gulten Soycan, Clemence Dine, Angelika Kristek, Sarah N. S. Alsharif, Volker Behrends, Alison Lovegrove, Peter R. Shewry, Jeremy P. E. Spencer

**Affiliations:** ^1^ Department of Food and Nutritional Sciences School of Chemistry, Food and Pharmacy University of Reading Reading UK; ^2^ Health Sciences Research Centre University of Roehampton London UK; ^3^ Rothamsted Research Harpenden UK

**Keywords:** absorption, avenanthramide, metabolism, oat bran, phenolic acid, whole grains

## Abstract

**Scope:**

Wholegrain has been associated with reduced chronic disease mortality, with oat intake particularly notable for lowering blood cholesterol and glycemia. To better understand the complex nutrient profile of oats, we studied urinary excretion of phenolic acids and avenanthramides after ingestion of oat bran in humans.

**Methods and results:**

After a 2‐d (poly)phenol‐low diet, seven healthy men provided urine 12 h before and 48 h after consuming 60 g oat bran (7.8 μmol avenanthramides, 139.2 μmol phenolic acids) or a phenolic‐low (traces of phenolics) control in a crossover design. Analysis by ultra‐high performance liquid chromatography (UPLC)–MS/MS showed that oat bran intake resulted in an elevation in urinary excretion of 30 phenolics relative to the control, suggesting that they are oat bran‐derived. Mean excretion levels were elevated between 0–2 and 4–8 h, following oat bran intake, and amounted to a total of 33.7 ± 7.3 μmol total excretion (mean recovery: 22.9 ± 5.0%), relative to control. The predominant metabolites included: vanillic acid, 4‐ and 3‐hydroxyhippuric acids, and sulfate‐conjugates of benzoic and ferulic acids, which accounted collectively for two thirds of total excretion.

**Conclusion:**

Oat bran phenolics follow a relatively rapid urinary excretion, with 30 metabolites excreted within 8 h of intake. These levels of excretion suggest that bound phenolics are, in part, rapidly released by the microbiota.

## 1 Introduction

Increasing the daily intake of wholegrain cereals by 90 g has been associated with reduction in mortality from cardiovascular disease by 27%, total cancer by 15%, respiratory disease by 22%, diabetes by 51%, and infectious diseases by 26%, as indicated by recent meta‐analysis of 45 prospective studies.^1^ Human intervention studies have to date largely focused on wholegrain oats (*Avena sativa* L.), with meta‐analyses establishing that regular oat intake lowers blood cholesterol^2^, ^3^ and improves insulin sensitivity and postprandial glycemic control.^4^ Although oats only account for 1% of world grain production, they, unlike more widely consumed grains, are almost exclusively consumed as wholegrains and therefore a rich dietary source of high quality proteins, minerals, vitamins, soluble β‐glucan fiber, and phenolic compounds (i.e. phenolic acids and avenanthramides), all of which are concentrated in the outer bran layers.^5^

β‐glucan is, at least in part, responsible for the health benefits of oats,^2^, ^3^, ^4^ while there is limited in vitro evidence that avenanthramides^6^ and phenolic acids^7^ may also promote beneficial cardiovascular physiology. Phenolic acid intake in Europe is on average 605 mg d^−1^, with the main dietary sources being coffee (75%), fruits (5.6%), and wholegrain products (5.5%).^8^ Wholegrain is the richest dietary source of ferulic acid, which has a mean intake of 38 mg d^−1^ in Europe,^8^ although a number of other phenolic acids and phenolic alkaloids, notably the avananthramides, are present in oats, either in the ‘‘free form’’, as soluble conjugates, or as insoluble bound forms (including ester linked to fiber).^9^ Understanding the urinary excretion (an indirect measure of absorption) of oat phenolic compounds following the dietary intake of whole oats or oat bran is a key prerequisite for determining which phenolic metabolites may mediate the health benefits of oats.

Previous data using oat phenolic extracts or wheat have indicated phenolics transfer to the circulation following intake,^10^, ^11^ while limited data exist for the urinary excretion of phenolics and other bioactive components from whole oats or oat bran at a dietary level. Notably, the intake of 150 mg of avananthramides (highly concentrated oat extract), led to detection of avenanthramides 2c, 2f, and 2p at nanomolar concentrations in plasma between 0.25 and 5 h, peaking at 2 h.^11^ While this study did not examine metabolism, data from studies in animals suggest that avenanthramide 2c is metabolized to avenanthramide 2f, dihydroavenanthramide 2f and 2c, four hydroxycinnamic acids, and 5‐hydroxyanthranilic acid following oral intake.^12^ Furthermore, intake of 94 g wholegrain wheat bread, containing 87 mg of ferulic acid or aleurone‐enriched white bread led to the appearance of ferulic acid‐sulfate, dihydroferulic‐sulfate, hippuric acid, and two hydroxyhippuric acids in plasma, with the former two reaching peak plasma concentrations of 84 nm and 9 nm at 1 and 7 h after intake, respectively,^10^ along with 12 other phenolic acid metabolites in 48 h urine, suggesting that ferulic acid is subject to extensive metabolism and that some fiber‐linked ferulic acid is released later during transit through the gastrointestinal tract through action of the microbiota.^10^

The aim of our study was to examine the urinary excretion of avenanthramides and phenolic acids following intake of 60 g oat bran. Specifically, the study focused on three objectives; (1) to establish peak urinary excretion intervals of phenolic acids and avenanthramides; (2) to determine the temporal nature of oat phenolic release from the bound state in the gastrointestinal tract; and (3) to identify and quantify the range of phenolic metabolites derived from the oat phenolics using comprehensive UPLC–MS/MS methods.

## 2 Experimental Section

### 2.1 Chemicals and Reagents

Avenanthramide 2c, avenanthramide 2f, avenanthramide 2p, 2,4‐dihydroxy benzoic acid, *p*‐coumaric acid, caffeic acid, isoferulic acid, syringic acid, salicylic acid, o‐coumaric acid, vanillic acid, syringaldehyde, ferulic acid, sinapic acid, 4‐hydroxybenzaldehyde, 4‐hydroxybenzoic acid, vanillin, protocatechuic acid, isovanillic acid, gallic acid, homovanillic acid, hippuric acid, dihydroferulic acid, dihydrocaffeic acid, 4‐hydroxyphenylacetic acid, and 2‐hydroxyhippuric acid were obtained from Sigma–Aldrich (see Supporting Information Table S1 for IUPAC names). Dihydroferulic acid‐4‐*O*‐glucuronide, isoferulic acid‐3‐*O*‐sulfate, ferulic acid‐4‐*O*‐glucuronide, dihydroxybenzoic acid‐3‐*O*‐glucuronide, and 5‐hydroxyanthranilic acid were obtained from Toronto Research Chemicals Inc. 4‐Hydroxyhippuric acid and 3‐hydroxyhippuric acid were purchased from Enamine (see Supporting Information Table S1 for IUPAC names). All solvents were HPLC grade and were obtained from Sigma–Aldrich or Fisher Scientific. While we acknowledge that avenanthramides are phenolic alkaloids, the terms phenolics is used throughout the paper to include both avenanthramides and phenolic acids.

### 2.2 Extraction and Analysis of Oat Bran Phenolics

Soluble and bound phenolic fractions were extracted from oats using an established method,^13^ with addition of hexane defatting steps adapted from^14^ and preservation of phenolics during alkali hydrolysis using ascorbate and EDTA adapted from.^15^ Phenolic acids and avenanthramides were separated using an Agilent 1100 series HPLC equipped with a Kinetex biphenyl column (100 Å 250 × 4.6 mm, 5μm; Phenomenex) with a Security Guard ultra‐biphenyl cartridge (Phenomenex). Sample injection volume was 20 μL, the flow rate 1 mL min^−1^, and mobile phases consisted of 0.1% formic acid v/v in water (solvent A) and 0.1% formic acid v/v in methanol (solvent B). The solvent gradient consisted of 5% B at 0 min, 25% B at 20 min, 26% B at 25 min, 35% B at 30 min, 36% B at 40 min, 70% B at 53 min, 95% B at 56 min, 95% B at 61 min, 5% B at 62 min, and 5% B at 65 min. The absorbance was recorded at 254, 280, and 320 nm and quantification was based on 12‐point linear calibration curves (mean *R* ^2^ > 0.994) and as a ratio to the internal standard (i.e. 3,5‐dichloro‐4‐hydroxybenzoic acid) to account for losses during extraction.

### 2.3 Study Design

Seven healthy men aged 25–62 years were recruited from the local community. Exclusion criteria were as follows: recent (last 3 months) use of antibiotics, flu vaccination, or dietary supplements. All participants gave written informed consent prior to study commencement and the study was performed at the Hugh Sinclair Unit of Human Nutrition, University of Reading (UK) between July 2014 and September 2014. The study was approved by the University of Reading Research Ethics Committee (Reference number: 31/15), followed the principles of the Declaration of Helsinki and was registered on ClinicalTrials.gov under NCT02574039.

**Figure 1:**
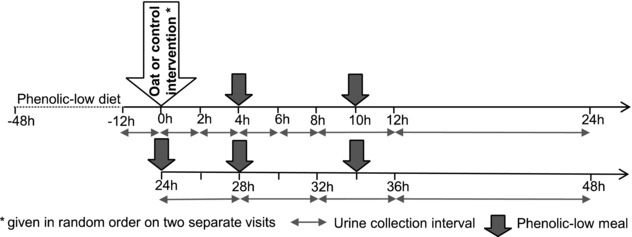
Study design overview.

The study was designed as a non‐blinded, randomized, controlled trial, where participants attended two experimental visits that were identical with the exception of the two study meals: (1) test intervention consisting of 60 g oat bran porridge, 200 mL semi‐skimmed milk, and 100 mL water, microwaved (2 min); or (2) a control consisting of 100 g white bread, 14 g butter, golden syrup, one boiled egg, and 200 mL semi‐skimmed milk; given in random order. Participants arrived at the Nutrition Unit at 8 am or 9 am to consume the study meal within 10 min. Urine was collected at 11 specific intervals relative to study meal consumption: −12 to 0 h (i.e. baseline), 0 to 2 h, 2 to 4 h, 4 to 6 h, 6 to 8 h, 8 to 12 h, 12 to 24 h, 24 to 28 h, 28 to 32 h, 32 to 36 h, and 36 to 48 h (see **Figure** 1). Collected urine was kept on ice packs, the excretion volume measured, and aliquots were stored at −80 °C with and without 5% formic acid acidification. During the 48 h urine collection period, participants consumed low‐phenolic meals provided to them (lunch: a white bread cheese sandwich and toffee yoghurt; dinner: macaroni cheese pasta, white bread roll, and crème brulée; breakfast: white toast with butter and golden syrup, a boiled egg, and glass of milk), while drinking water ad libitum. Prior to each experimental visit, participants followed a diet low in (poly)phenols for 48 h (i.e. no fruits, vegetables, wholegrains, pulses, spices, herbs, nuts, seeds, chocolate, tea, and coffee), and attended an overnight fast after having consumed a low‐(poly)phenol dinner (a cheese pasta bake, white bread roll, and crème brulée). Compliance to the dietary restrictions was assessed using food intake diaries and questionnaires. One volunteer was excluded from the final dataset, due to non‐compliance to the dietary restrictions (i.e. volunteer reported intake of (poly)phenol‐rich foods and baseline and post‐control urine contained high levels of phenolics).

### 2.4 Solid Phase Extraction

Phenolic acids were extracted from urine using a validated method^16^ with minor modifications. Briefly, 1 mL of urine was spiked with an internal standard (i.e. 3,5‐dichloro‐4‐hydroxybenzoic acid) and subsequently extracted using SPE cartridges (Strata‐X columns 500 mg 6mL^−1^; Phenomenex). These were washed with 12 mL of 0.1/99.9 v/v hydrochloric acid/water, dried for 30 min under vacuum, soaked in 0.1/99.9 v/v hydrochloric acid/methanol for 10 min, and eluted into glass vials with 7 mL 0.1/99.9 v/v hydrochloric acid/methanol. Samples were evaporated to complete dryness under speedvac at room temperature. The dried samples were resuspended in 250 μL of mobile phase (0.1/5/94.9, v/v/v, formic acid/methanol/water) by 30 s vortexing, 15min ultrasound sonicating, and 1 h shaking. Samples were stored at −80 °C until analysis. For phenolic acids and metabolites the method has a mean ± SD extraction efficiency of 88.3 ± 17.8% as previously reported,^16^ while we established extraction efficiencies of 102, 97, and 57% for avenanthramide A, B, and C, respectively.

### 2.5 UPLC–MS/MS Analysis

The UPLC–ESI–MS/MS system consisted of an Aquity UPLC Hclass (Waters) coupled to a Xevo TQ‐S micro ESI mass spectrometer (Waters) operated using MassLynx software (V4.1, Waters Inc, USA). Compound separation was achieved using an Aquity UPLC HSS T3 1.8μm column (2.1 × 100 mm) attached to a Van guard precolumn of the same material and pore size, maintained at 45 °C with a flow of 0.65 mL min^−1^ and a sample injection volume of 2 μL. The mobile phase consisted of 0.1/99.9 v/v formic acid/water (A) and 0.1/99.9 v/v formic acid/acetonitrile (B); and a mobile phase gradient consisting of: 1% B at 0 min, 1% B at 1 min, 30 % B at 10 min, 95 % B at 12 min, 95% B at 13 min, 1% B at 13.10 min, 1% B at 16 min. A scheduled multiple reaction monitoring (sMRM) method was developed by syringe infusion of 34 analytical standards (see section Chemicals and Reagents and Supporting Information Table S1) to determine sMRM transitions, optimal sMRM modes (i.e. negative or positive) and collision energies (Supporting Information Table S2).

Regarding phase II metabolites, while four authentic standards were used for the sMRM method (i.e. dihydroferulic acid‐4‐*O*‐glucuronide, isoferulic acid‐3‐*O*‐sulfate, ferulic acid‐4‐*O*‐glucuronide, and dihydroxybenzoic acid‐3‐*O*‐glucuronide), further putative glucuronide or sulfate conjugated phenolic acid metabolites and feruloyl glycine were added to the sMRM method, even though analytical standards were not commercially available. For these, retention times and sMRM transitions were tentatively identified by injecting a pooled extract of urine (i.e. using urine collected after oat bran intake from all *n* = 7 participants and during all ten post‐intake time intervals). sMRM transitions were taken from the literature^17^, ^18^, ^19^, ^20^ or derived from the fragmentation pattern of the phenolic acid aglycones by adding the *m/z* of glucuronide (i.e. 176) or sulfate (i.e. 80) to the precursor ion and including the appropriate MS/MS fragment. Furthermore, 113 *m/z*, a common fragment of glucuronic acid, was added as an MS/MS fragment for glucuronide conjugates.^19^ Collision energies were optimized to a limited extent by injecting the pooled urine extract three times at collision energies −11, −17, and −21 and the best one was selected for each sMRM transition. In the final sMRM, a total of 74 potential compounds were targeted and statistical comparison between the oat and control intervention was used to identify those which are oat bran‐derived phenolics. Identification of the phenolic metabolites for which standards were available was based on their retention times and the major sMRM ion transitions of the standards, while the tentative identification of phenolic metabolites for which analytical standards were not available was based on 3–6 sMRM ion transitions (except for benzoic acid‐sulfate which only has two sMRM ion transitions as reported in the literature^20^) (Supporting Information Table S2).

Quantification was established using the most intense sMRM transition and 11–14 point calibration curves of analytical standards (Supporting Information Table S2). Where pure standards were not available, quantification was conducted relative to standard curves of compounds with similar structures (e.g. the calibration curve of isoferulic acid‐3‐*O*‐sulfate was used to quantify all tentatively identified sulfate metabolites). The LOD and LOQ were established for each compound as the concentrations of peaks with S/N of 3 and 10, respectively, and were lowest for avenanthramide 2p (0.8 and 2.7 nm, respectively) and highest for vanillin or hydroxyphenylacetic acid‐*O*‐glucuronide (35.0 and 116.7 nm, respectively; Supporting Information Table S2). Blanks and quality controls were run every ten injections. Validation parameters indicate that the method had good precision and repeatability (interday variability: 9.1%; interday variability: 13.7%) and good linearity (linear regression coefficient range: 0.990–1.000; Supporting Information Table S2).

Sample acidification using 5% formic acid did not significantly affect phenolic compound peak areas (established in *n* = 3 volunteers; data not shown) and therefore non‐acidified urine was used for the complete analysis.

### 2.6 Statistical Analysis

A two‐factor repeated‐measurement linear mixed model was fitted to analyze hourly urinary excretion data. The model included participants nested within time as a random effect and baseline hourly urinary excretion, intervention, time, and interaction as fixed effects. When the model showed a significant interaction effect, post‐hoc analysis with Tukey–Kramer adjustment was performed. Data are presented as mean ± SEMs. *p*‐values < 0.05 were considered statistically significant, and statistical analysis was performed by using R programming language version 3.1.2 (R Development Core Team, 2014).

## 3 Results

### 3.1 Phenolic Composition of the Oat Bran Intervention

Twelve phenolic compounds were detected in the oat bran used for the trial, with eight phenolic acids being present in both soluble and bound fractions, three avenanthramides only present in the soluble fraction and vanillin only in the bound fraction (**Table** 1). The intervention diet (60 g oat bran) contained 28.6 mg total phenolics (24% in the soluble fraction), with ferulic acid being the predominant phenolic acid (16.8 mg) followed by *p*‐coumaric acid (3.3 mg) and the three avenanthramides collectively amounting to 2.5 mg. Although the control meal and all other meals consumed during the 48 h study periods were not analyzed, they consisted of white wheat bread and pasta and would therefore be expected to contain only low amounts of phenolic acids.^21^

**Table 1:** Content and composition of phenolics in 60 g oat bran.a)

	Fraction		
	Conjugated and free	Bound	% Conjugated and free
*Avenanthramides*			
Avenanthramide 2c	0.8 mg [2.5 μmol]	ND	100
Avenanthramide 2f	1.0 mg [3.1 μmol]	ND	100
Avenanthramide 2p	0.7 mg [2.2 μmol]	ND	100
*Hydroxycinnamic acids*			
Ferulic acid	2.0 mg [10.1 μmol]	14.8 mg [76.4 μmol]	12
*p*‐Coumaric acid	0.4mg [2.3 μmol]	3.0 mg [18.1 μmol]	11
Caffeic acid	0.1mg [0.4 μmol]	1.3 mg [7.0 μmol]	5
Sinapic acid	0.9 mg [3.8 μmol]	1.2 mg [5.5 μmol]	41
*Hydroxybenzoic acids*			
4‐Hydroxybenzoic acid	0.2 mg [1.8 μmol]	0.3 mg [1.9 μmol]	48
Vanillic acid	0.4 mg [2.4 μmol]	0.4 mg [2.2 μmol]	53
Syringic acid	0.4 mg [2.1 μmol]	0.3 mg [1.5 μmol]	57
*Benzaldehydes*			
4‐Hydroxybenzaldehyde	0.2 mg [1.4 μmol]	0.1 mg [1.1 μmol]	56
Vanillin	ND	0.2 mg [1.3 μmol]	0
Total	7.0 mg [32.1 μmol]	21.6 mg [115.0 μmol]	24

^a)^ 60 g of oat bran porridge made with 200 mL semi‐skimmed milk and 100 mL water. Heated by microwave.

ND, not detected.

### 3.2 Identification of Oat Bran‐Derived Phenolic Compounds in Urine

A wide range of phenolic compounds were detected in urine at baseline and after the control meal intake (Supporting Information Table S3). However, the hourly excretion of 30 individual phenolic compounds was elevated following intake of oat bran as compared to the control intervention (significant mixed model *p*‐value of intervention and/or time*intervention; Supporting Information Table S3), suggesting that they are derived from the ingested oat bran. These phenolics were identified as avenanthramides 2f and 29 phenolic acids (nine aglycones, four glycine conjugates, ten sulfate conjugates, and seven glucuronide conjugates; Supporting Information Table S2). Excretion of hippuric acid, the dominant phenolic acid in human urine, was not statistically higher after the oat bran intervention relative to the control and was thus not identified as an oat bran‐derived metabolites (mixed model time*intervention interaction *p*‐values: 0.36; Supporting Information Table S4). In total, 44 targeted phenolic compounds were statistically similar between interventions (Supporting Information Table S4).

### 3.3 Hourly Urinary Excretion of Oat Phenolic Compounds

**Figure 2:**
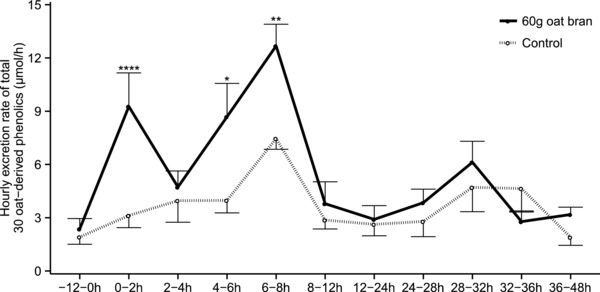
Urinary excretion rate of total 30 oat bran‐derived phenolic compounds after intake of 60 g oat bran or a control meal in healthy men (μmol h^−1^). Data are reported as mean ± SEM and were analyzed by two‐factor repeated measurement linear mixed model with time and intervention as the two factors [significant effect of intervention (*p* = 0.005), time (*p* = 3×10^−13^), and time and intervention interaction (*p* = 5×10^−6^)]. Post‐hoc analysis with Tukey–Kramer adjustment was performed and *p*‐values are indicated as follow: * *p* < 0.05; ** *p* < 0.01; **** *p* < 0.0001.

**Figure 3:**
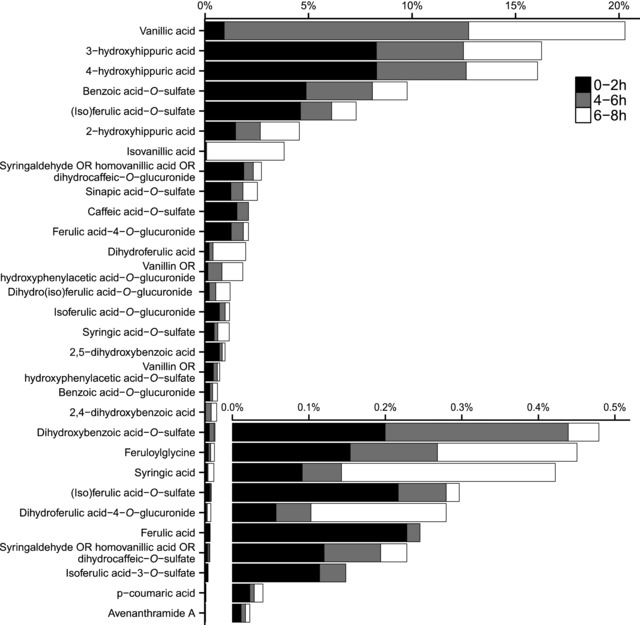
Proportions of individual oat bran‐derived phenolics relative to total excretion levels at 0–2 h, 4–6 h, and 6–8 h. Excretion level differences between the oat and control interventions were used to calculate percentages.

At baseline, the mean hourly excretion of the total excretion of 30 phenolic compounds prior to oat bran and control interventions was 2.4 ± 0.6 μmol h^−1^ and 1.9 ± 0.4 μmol h^−1^, respectively (**Figure** 2). Following intervention, the total hourly phenolic excretion significantly increased at 0–2 h (oat: 9.3 ± 1.9 μmol h^−1^, control: 3.1 ± 0.7 μmol h^−1^; post‐hoc *p*‐value <0.0001), returned to a similar level as control at 2–4 h (oat: 4.7 ± 0.9 μmol h^−1^, control: 4.0 ± 1.2 μmol h^−1^; post‐hoc *p*‐value >0.05) and peaked again between the 4–6 h period (oat: 8.7 ± 1.9μmol h^−1^, control: 4.0 ± 0.7μmol h^−1^; post‐hoc *p*‐value < 0.05) and 6–8h (oat: 13.7 ± 1.2 μmol h^−1^, control: 7.5 ± 0.6 μmol h^−1^; post‐hoc *p*‐value < 0.01) before returning to a similar level to the control intervention from 8 to 48 h (post‐hoc *p*‐value > 0.05; Figure 2). **Figure** 3 shows the oat bran induced increase in excretion of the individual 30 oat bran‐derived phenolics proportional to the total excretion at 0–2 h, 4–6 h and 6–8 h, relative to control. Vanillic acid, 3‐hydroxyhippuric acid, 4‐hydroxyhippuric acid, benzoic acid‐*O*‐sulfate and ferulic acid‐ *O*‐sulfate were the predominant oat bran‐derived phenolics excreted, accounting collectively for more than two thirds of the total excretion (i.e. 20.3, 16.3, 16.1, 9.8, and 7.3%, respectively). (Iso)ferulic acid‐*O*‐sulfate was predominant during the early 0–2 h peak, vanillic acid was almost exclusively excreted during the late 4–8 h period, while excretion of hydroxyhippuric acids and benzoic acid‐*O*‐sulfate was biphasic, with early and late peaks (Figure 3). Ferulic acid, *p*‐coumaric acid, and avenanthramides 2p (i.e. the most abundant phenolic compounds in oats) accounted only for small percentages of the total excreted phenolics (i.e. 0.21, 0.04, and 0.02%, respectively; Figure 3) and avenanthramide 2f and 2c were not detected, suggesting that these dietary forms are subject to extensive metabolism.

The mean absolute urinary excretion of the 30 oat bran‐derived phenolics from 0–8 h was 70.7 ± 8.1μmol after consumption of oat bran and 37.1 ± 4.4 μmol after the control intervention (**Table** 2). Hence, on average 22.9 ± 5.0% of the ingested dose (i.e. 147μmol phenolics in 60 g oat bran; Table 1) was recovered in the 0–8 h urine and there was large inter‐individual variability in absorption that ranged from 3.7 to 41.2 % (Table 2). The mean percentage recovery of metabolites was lowest for the avenanthramide 2p (0.3%), intermediate for hydroxycinnamic acids (6.8–10.7%), and highest for hydroxybenzoic acids (14.5–159.2%; Table 2).

**Table 2:** 0–8 h absolute urinary excretion (μmol) of oat bran‐derived phenolics and recovery of ingested dose (%).

	Mean ± SEM urinary excretion 0–8 h [μmol]			Recovery of ingested dose [%]	
Excreted phenolics	Control	Oat bran	Difference	Mean ± SEM	Lowest/highest absorber	Oat parent compounds (Ingested dose)
*Avenanthramide 2p*	0.006 ± 0.001	0.012 ± 0.003	0.006 ± 0.003	0.3 ± 0.1	0 /0.7	Avenanthramide 2p [2.2 μmol]
*Hydroxycinnamic acids*						
Ferulic acid metabolitesa)	4.1 ± 0.4	10 ± 0.8	5.9 ± 0.7	6.8 ± 0.8	4.9 /9.3%	Ferulic acid [86.5 μmol]
Caffeic acid‐*O*‐sulfate	0.4 ± 0.2	1.2 ± 0.4	0.8 ± 0.5	10.7 ± 7	−15.6 /34.9	Caffeic acid [7.4 μmol]
Sinapic acid‐*O*‐sulfate	0.5 ± 0.1	1.5 ± 0.2	1 ± 0.2	10.6 ± 1.6	4.9 /15	Sinapic acid [9.3 μmol]
*Hydroxybenzoic acids*						
(Iso)vanillic acid	6.1 ± 2.5	13.5 ± 3.1	7.3 ± 2.5	159.2 ± 53.8	−80.5 /308.3	Vanillic acid [4.6 μmol]
Benzoic acid‐*O*‐sulfate/glucuronide	7.1 ± 2	10.3 ± 2.7	3.2 ± 1	85.2 ± 26	8.1 /176.1	4‐hydroxybenzoic acid [3.7 μmol]
Syringic acid‐*O*‐sulfate	0.4 ± 0.1	0.9 ± 0.2	0.5 ± 0.1	14.5 ± 4.1	1.5 /30.5	Syringic acid [3.6 μmol]
Total 30 phenolics	37.1 ± 4.4	70.7 ± 8.1	33.7 ± 7.3	22.9 ± 5	3.7 /41.2	Total 12 phenolics [147.1 μmol]

^a)^Sum of ferulic, isoferulic, dihydroferulic, and dihydroisoferulic acids: aglycones, glucuronides, or sulfates.

## 4 Discussion

The present randomized controlled trial is, to our knowledge, the first to have examined the excretion of phenolic metabolites following intake of dietary amounts of oat bran as a whole food over a 48 h timeframe and complements previous studies that examined the metabolism and bioavailability of phenolics following intake of an oat avenanthramide extract at a high‐dose^11^ or following intake of wheat.^10^ Oat bran intake resulted in elevated urinary excretion of 30 phenolic compounds at 0–2 h and 4–8 h, with sulfate or glycine conjugated benzoic acids being the major metabolites together with ferulic acid*‐O*‐sulfate, and the total amounts of phenolic compounds recovered in urine amounting to 22.9 ± 5.0% of the ingested phenolic dose. These results suggest that a high proportion of oat phenolics are bioavailable as a wide range of metabolites with absorption occurring both in the small intestine and then in the large intestine within eight hours of consumption.

Analysis of the utilized oat bran detected nine phenolic acids amounting to 434 μg g^−1^ and three avenanthramides amounting to 41 μg g^−1^ (Table 1). These levels are comparable to four previous studies that analyzed wholegrain oats or oat bran, identifying up to ten phenolic acids at levels ranging from 273 to 874 μg g^−1^ and three avenanthramides at levels ranging from 13 to 116 μg g^−1^.^9^, ^22^, ^23^, ^24^ The relatively wide range in the reported contents and compositions of phenolics can be explained by the different oat products analyzed (i.e. commercial or non‐commercial varieties, bran or wholegrain, and hulled or de‐hulled), and by the different extraction and analytical methods used for their analysis. Although other cereals may be richer sources of phenolic compounds (for example, Mattila et al. found 6.8‐fold and 6.3‐fold more total phenolic compounds in wheat and rye bran, respectively, than in oat bran^23^), oats are particularly interesting because of the relatively high proportions of phenolics in free and conjugated forms (24% in our oat bran and from 34 to 62% in non‐commercial hulled wholegrain oats,^9^ whereas wheat has less than 18%^21^ and wheat bran cereals only 8%^18^) and thus phenolic components from oats may have a higher bio‐accessibility in comparison to other cereals.

We observed high background excretion of all 30 oat bran‐derived phenolic compounds at baseline and in the control arm (Supporting Information Table S3; Figure 2), even though volunteers followed a 48 h low‐phenolic diet. A high background phenolic excretion has previously been described in the control groups of other studies of (poly)phenol bioavailability after following 2 d^25^ or 7 d^26^ low phenolic dietary restrictions. These phenolic compounds may originate from the relatively low amounts of phenolics consumed in white bread and pasta during the dietary restrictions^21^ and from the metabolism of other dietary components such as aromatic amino acids.^27^ Despite this high background excretion, our data indicate that intake of oat bran leads to urinary excretion of 30 phenolic acid compounds which peak early at 0–2 h, and again later between 4–8 h (Figure 2). The early appearance of metabolites suggests that the absorption of free phenolics from the oat bran occurs in the upper gastrointestinal tract with esterified conjugates being hydrolyzed by esterase activity in the intestinal mucosa,^28^ followed by the transfer of free phenolics across the intestinal epithelium through passive diffusion or via transporters.^29^ Although we hypothesized that phenolics in the bound fraction would appear late in the urine, due to a requirement for gut microbiota to cleave covalent linkages between phenolics and fiber, the second peak of excretion was completed by 8 h, suggesting that the release and absorption occurred more rapidly (Figure 2). This agrees with previous studies, showing that bound phenolics may reach the colon and undergo microbial fermentation within 4 h of oat bran intake. Indeed, in fasted volunteers (which was true in our study) a mean mouth‐to‐cecum transit time can be as rapid as 2.3 h^30^ and in vitro fermentation of wheat bran with human microbiota shows that digestion by microbial esterase and xylanase starts within 2 h resulting in most fiber‐bound phenolics being released within 6 h.^31^

The urinary recovery of ingested phenolics was on average 22.9 ± 5.0% in the first 8 h after the oat bran intake, relative to the control (Table 2). Previous non‐controlled studies reported lower mean recoveries in 24 h urine of 4 ± 1, 8 ± 2, and 3 ± 1% following ingestion of phenolics in wholegrain wheat bread,^10^ aleurone‐enriched wheat bread,^10^ or wheat bran cereals,^18^ respectively, but a higher mean recovery of 29 ± 4% following ingestion of phenolics in instant coffee.^17^ This wide variation in phenolic bioavailability may partly be explained by differences in the bioaccessibility of phenolics within the food matrix. While phenolic acids in coffee are conjugated to quinic acids and are relatively water soluble,^17^ the oat bran used here had 24% soluble phenolic acids (Table 1) and wheat bran cereals only 9%.^18^ However, such direct comparisons of recoveries between studies may be of limited value due to the non‐controlled design of previous studies,^10^, ^17^, ^18^ and differences in the methods used to analyze phenolic acids in the urine and food samples including the number of metabolites targeted with the LC–MS/MS methods.

Possible pathways for the metabolism of 12 ingested oat phenolic compounds into the 33 excreted urinary compounds are provided in Supporting Information Figure S1 and based on metabolic pathways previously described in the literature.^17^, ^25^, ^32^, ^33^ Notably, the major excreted compounds are downstream in the metabolic pathways (Figure 3 and Supporting Information Figure S1) and therefore products of extensive metabolism by endogenous and colonic microbial enzymes. Free forms of vanillic and isovanillic acids were the highest and seventh most highly excreted phenolics, respectively being mainly excreted in the late peak between 4–8 h after oat bran intake (Figure 3) and at levels totaling more than the total amount of vanillic acid that was ingested (mean ± SEM recovery: 159.2 ± 53.8%; Table 2). These data suggest that while only a limited amount of vanillic acid is absorbed directly in the small intestine, a larger proportion is absorbed following release by fermentation by the colonic microbiota with previous studies suggesting that it originated partly from the metabolism of avenanthramides and hydroxycinnamic acids.^25^, ^32^, ^34^, ^35^ Vanillic acid may be formed by β‐oxidation of ferulic acid in the liver, and isovanillic acid from methylation of caffeic acid to isoferulic acid followed by liver β‐oxidation (Supporting Information Figure S1).^25^, ^32^, ^34^, ^35^ 4‐, 3‐ and 2‐Hydroxyhippuric acids were also highly excreted, with their excretion following a biphasic pattern (early 0–2 h and late 4–8 h) after oat bran intake (Figure 3). While hydroxyhippuric acids are common flavonoid metabolites detected, for example, following intake of orange juice^26^ and cocoa,^36^ results from this oat trial and a recent wheat trial^10^ suggest that hydroxyhippuric acids are also important metabolites derived from wholegrain.

A study feeding 75–150 mg avenanthramides in an oat extract to humans detected nanomolar concentrations of circulating avenanthramides,^11^ while the present study detected only traces of the ingested 2.5 mg avenanthramides in urine. This may indicate that avenanthramides are bioavailable when ingested at a relatively high dose and that avenanthramides are mostly metabolized particularly to hydroxycinnamic acids during their passage through the gastrointestinal tract and into the circulation (Table 2). Hydroxycinnamic acids, in turn, undergo reduction, methylation, sulfation, or glucuronidation, and are also metabolized to smaller hydroxybenzoic acids (Table 2, Supporting Information Figure S1). Chlorogenic acid and caffeic acid aglycones in coffee have a similar metabolic fate to avenanthramides and hydroxycinnamic acids in cereals. A study in ileostomy patients showed that although 33% of chlorogenic acid and 95% of caffeic acid were absorbed in the small intestine, only traces of chlorogenic acid and 11% of caffeic acid of the ingested dose were excreted in the urine.^37^ Avenanthramides were also metabolize to hydroxyanthralinic acid and dihydroavenanthramides as reported in a study feeding mice with a high dose of 200 mg kg^−1^ avenanthramide 2c^12^; however, the present study did determine these two compounds because of poor ionization of the former and unavailability of sMRM parameters for the latter.

In conclusion, oats are a popular health food and to our knowledge the present study is the first to study the metabolic fate of oat avenanthramides and phenolic acids when ingested at a dietary dose and as a whole food. Our data suggest that oat bran phenolics are more bioavailable than previous studies reported^10^, ^17^, ^18^ with 22.9 ± 5.0% of the ingested dose being excreted in urine during 8 h following intake in the form of 30 different phenolics or their metabolites. The data showed that benzoic acid derivatives, and in particular (iso)vanillic acid, and three isoforms of hydroxyhippuric acids, accounted for a high proportion of the excreted compounds, together with ferulic acid*‐O*‐sulfate. While the present study is an important contribution to the existing literature, future work is required to verify tentatively identified phenolic metabolites against analytical standards, to establish the detailed pharmacokinetics and circulating concentrations of these oat bran‐derived phenolic compounds, and to determine their biological activities and contributions to the health benefits of a diet rich in oats.

**Abbreviations**
sMRM: scheduled multiple reaction monitoring
UPLC: ultra‐high performance liquid chromatography

## Conflict of Interest

The authors have declared no conflict of interest.

## Supporting information

Supporting Information Figure 1 – Possible metabolic pathways on how the twelve ingested oat phenolic compounds are metabolized into 30 excreted urinary compounds.
Supporting Information Table 1 – Common and IUPAC names of phenolic compounds
Supporting Information Table 2 – sMRM transitions, parameters and detection limits of 30 identified oat bran‐derived phenolics
Supporting Information Table 3 – Urinary excretion rate per hour of oat bran‐derived phenolic compounds after intake of 60g oat bran or a control meal in six healthy men (nmol h^−1^)
Supporting Information Table 4 – sMRM transitions, parameters and detection limits of non oat bran‐derived phenolics
Supporting Information Table 5 – Urinary excretion rate per hour of non oat bran‐derived phenolic compounds after intake of 60g oat bran or a control meal in six healthy men (nmol h^−1^)
