# Supplementary material for: Excretion of Avenanthramides, Phenolic Acids and their Major Metabolites Following Intake of Oat Bran
Source: Mol Nutr Food Res. 2017 Dec 29;62(2):1700499. doi: 10.1002/mnfr.201700499 (PMC5836716; doi:10.1002/mnfr.201700499)
Supplement: Supplementary file 1 — Supporting Information Figure 1 – Possible metabolic pathways on how the twelve ingested oat phenolic compounds are metabolized into 30 excreted urinary compounds. Supporting Information Table 1 – Common and IUPAC names of phenolic compounds Supporting Information Table 2 – sMRM transitions, parameters and detection limits of 30 identified oat bran‐derived phenolics Supporting Information Table 3 – Urinary excretion rate per hour of oat bran‐derived phenolic compounds after intake of 60g oat bran or a control meal in six healthy men (nmol h−1) Supporting Information Table 4 – sMRM transitions, parameters and detection limits of non oat bran‐derived phenolics Supporting Information Table 5 – Urinary excretion rate per hour of non oat bran‐derived phenolic compounds after intake of 60g oat bran or a control meal in six healthy men (nmol h−1) [file MNFR-62-na-s001.docx]

**Supporting Information Figure 1 – Possible metabolic pathways on how the twelve ingested oat phenolic compounds are metabolized into 30 excreted urinary compounds.** The five top metabolites are highlighted with a blue background. COMT, catechol-O-methyl transferase, β-Ox, β-oxidase, GT, glucuronic acid transferase; ST, sulfate transferase; CoA, co-enzyme A

**Supporting Information Table 1 – Common and IUPAC names of phenolic compounds**

|  |  |
| --- | --- |
| **Common Name** | **IUPAC Name** |
| *List of available analytical standards* | |
| 2,4-dihydroxy benzoic acid | 2,4-dihydroxybenzoic acid |
| 2,5-dihydroxy benzoic acid | 2,5-dihydroxy benzoic acid |
| 2-hydroxyhippuric acid | 2-[(2-hydroxybenzoyl)amino]acetic acid |
| 3-hydroxyhippuric acid | 2-[(3-hydroxybenzoyl)amino]acetic acid |
| 4-hydroxybenzaldehyde | 4-hydroxybenzaldehyde |
| 4-hydroxybenzoic acid | 4-hydroxybenzoic acid |
| 4-hydroxyhippuric acid | 2-[(4-hydroxybenzoyl)amino]acetic acid |
| 4-hydroxyphenylacetic acid | 2-(4-hydroxyphenyl)acetic acid |
| 5-hydroxyanthranilic acid | 2-amino-5-hydroxybenzoic acid |
| Avenanthramide 2c | 2-[[(E)-3-(3,4-dihydroxyphenyl)prop-2-enoyl]amino]-5-hydroxybenzoic acid |
| Avenanthramide 2f | 5-hydroxy-2-[[(E)-3-(4-hydroxy-3-methoxyphenyl)prop-2-enoyl]amino]benzoic acid |
| Avenanthramide 2p | 5-hydroxy-2-[[(E)-3-(4-hydroxyphenyl)prop-2-enoyl]amino]benzoic acid |
| Caffeic acid | (E)-3-(3,4-dihydroxyphenyl)prop-2-enoic acid |
| Dihydrocaffeic acid | 3-(3,4-dihydroxyphenyl)propanoic acid |
| Dihydroferulic acid | 3-(4-hydroxy-3-methoxyphenyl)propanoic acid |
| Dihydroferulic acid-4-*O*-glucuronide | (2S,3S,4S,5R,6S)-6-[4-(2-carboxyethyl)-2-methoxyphenoxy]-3,4,5-trihydroxyoxane-2-carboxylic acid |
| Dihydroxybenzoic acid-3-*O*-glucuronide | (2S,3S,4S,5R,6S)-6-(5-carboxy-2-hydroxyphenoxy)-3,4,5-trihydroxyoxane-2-carboxylic acid |
| Ferulic acid | (E)-3-(4-hydroxy-3-methoxyphenyl)prop-2-enoic acid |
| Ferulic acid-4-*O*-glucuronide | (2S,3S,4S,5R,6S)-6-[4-[(E)-2-carboxyethenyl]-2-methoxyphenoxy]-3,4,5-trihydroxyoxane-2-carboxylic acid |
| Gallic acid | 3,4,5-trihydroxybenzoic acid |
| Hippuric acid | 2-benzamidoacetic acid |
| Homovanillic acid | 2-(4-hydroxy-3-methoxyphenyl)acetic acid |
| Isoferulic acid | (E)-3-(3-hydroxy-4-methoxyphenyl)prop-2-enoic acid |
| Isoferulic acid-3-*O*-sulfate | (E)-3-[3-sulfooxy-4-(methoxy)phenyl]prop-2-enoic acid |
| Isovanillic acid | 3-hydroxy-4-methoxybenzoic acid |
| o-coumaric acid | (E)-3-(2-hydroxyphenyl)prop-2-enoic acid |
| p-coumaric acid | (E)-3-(4-hydroxyphenyl)prop-2-enoic acid |
| Protocatechuic acid | 3,4-dihydroxybenzoic acid |
| Salicylic acid | 2-hydroxybenzoic acid |
| Sinapic acid | (E)-3-(4-hydroxy-3,5-dimethoxyphenyl)prop-2-enoic acid |
| Syringaldehyde | 4-hydroxy-3,5-dimethoxybenzaldehyde |
| Syringic acid | 4-hydroxy-3,5-dimethoxybenzoic acid |
| Vanillic acid | 4-hydroxy-3-methoxybenzoic acid |
| Vanillin | 4-hydroxy-3-methoxybenzaldehyde |
| Tentatively identified oat-derived phenolics | |
| 3,4-dihydroxyhydrocinamic acid-*O*-glucuronide | (2S,3S,4R,5R,6S)-3-(5-(2-carboxyethyl)-2-hydroxyphenoxy)-4,5,6-trihydroxytetrahydro-2H-pyran-2-carboxylic acid |
| 4-hydroxyphenylacetic acid-*O*-glucuronide | (2S,4R,5R,6S)-3-(4-(carboxymethyl)phenoxy)-4,5,6-trihydroxytetrahydro-2H-pyran-2-carboxylic acid |
| Benzoic acid-*O*-glucuronide | (2S,3S,4R,5R,6S)-3-(4-carboxyphenoxy)-4,5,6-trihydroxytetrahydro-2H-pyran-2-carboxylic acid |
| Benzoic acid-O-sulfate | 4-carboxyphenyl sulfate |
| Caffeic acid-O-sulfate | (E)-4-(2-carboxyvinyl)-2-hydroxyphenyl sulfate |
| Dihydroferulic acid-O-glucuronide | (2S,3S,4R,5R,6S)-3-(4-(2-carboxyethyl)-2-methoxyphenoxy)-4,5,6-trihydroxytetrahydro-2H-pyran-2-carboxylic acid |
| Dihydroxybenzoic acid-O-sulfate | 4-carboxy-2-hydroxyphenyl sulfate |
| Feruloylglycine | 2-[[(E)-3-(4-hydroxy-3-methoxyphenyl)prop-2-enoyl]amino]acetic acid |
| Homovanillic acid-O-glucuronide | (2S,3S,4S,5R)-6-(4-(carboxymethyl)-2-methoxyphenoxy)-3,4,5-trihydroxytetrahydro-2H-pyran-2-carboxylic acid |
| Hydroxyphenylacetic acid-O-sulfate | 4-(carboxymethyl)phenyl sulfate |
| (Iso)ferulic acid-O-glucuronide | (2S,3S,4R,5R,6S)-3-(4-((E)-2-carboxyvinyl)-2-methoxyphenoxy)-4,5,6-trihydroxytetrahydro-2H-pyran-2-carboxylic acid |
| (Iso)ferulic acid-O-sulfate | 4-(2-carboxyethyl)-2-methoxyphenyl sulfate |
| Sinapic acid-O-sulfate | (E)-4-(2-carboxyvinyl)-2,6-dimethoxyphenyl sulfate |
| Syringaldehyde-O-glucuronide | (2S,3S,4R,5R,6S)-3-(4-formyl-2,6-dimethoxyphenoxy)-4,5,6-trihydroxytetrahydro-2H-pyran-2-carboxylic acid |
| Syringaldehyde-O-sulfate | 4-formyl-2-hydroxy-6-methoxyphenyl sulfate |
| Syringic acid-O-sulfate | 4-carboxy-2,6-dimethoxyphenyl sulfate |
| Vanillin-O-glucuronide | (2S,3S,4R,5R,6S)-3-(4-formyl-2-methoxyphenoxy)-4,5,6-trihydroxytetrahydro-2H-pyran-2-carboxylic acid |
| Vanillin-O-sulfate | 4-formyl-2-methoxyphenyl sulfate |
| Non oat-derived phenolics |  |
| 3,4-dihydrocaffeic acid | 3-(3,4-dihydroxyphenyl)propanoic acid |
| 4-hydroxybenzaldehyde | 4-hydroxybenzaldehyde |
| 4-hydroxyphenylacetic acid | 2-(4-hydroxyphenyl)acetic acid |
| 3-hydroxyphenylacetic acid | 2-(3-hydroxyphenyl)acetic acid |
| Dihydroisoferulic acid | 3-(3-hydroxy-4-methoxyphenyl)propanoic acid |
| Isoferulic acid | (E)-3-(3-hydroxy-4-methoxyphenyl)prop-2-enoic acid |
| o-coumaric acid | (E)-3-(2-hydroxyphenyl)prop-2-enoic acid |
| Salicylic acid | 2-hydroxybenzoic acid |
| Syringaldehyde | 4-hydroxy-3,5-dimethoxybenzaldehyde |
| Vanillin | 4-hydroxy-3-methoxybenzaldehyde |
| 4-hydroxybenzoic acid | 4-hydroxybenzoic acid |
| Caffeic acid | (E)-3-(3,4-dihydroxyphenyl)prop-2-enoic acid |
| Gallic acid | 3,4,5-trihydroxybenzoic acid |
| Hippuric acid | 2-benzamidoacetic acid |
| Homovanillic acid | 2-(4-hydroxy-3-methoxyphenyl)acetic acid |
| Protocatechuic acid OR acid | 3,4-dihydroxybenzoic acid |
| 3,5-dihydroxybenzoic | 3,5-dihydroxybenzoic |
| Salicylic acid-O-sulfate | 2-sulfooxybenzoic acid |
| Dihydroxybenzoic acid-O-sulfate | 3-carboxy-2-hydroxyphenyl sulfate |
| Coumaric acid-O-sulfate | (E)-3-(4-sulfonatooxyphenyl)prop-2-enoate |
| (iso)vanillic acid-O-sulfate | 4-carboxy-2-methoxyphenyl sulfate |
| Hippuric acid-O-sulfate | (2-(sulfooxy)benzoyl)glycine |
| Homovanillic acid-O-sulfate | 2-(3-methoxy-4-(sulfooxy)phenyl)acetic acid |
| Dihydroxyhydroycinamic acid-O-sulfate | 3-(2-carboxy-2,2-dihydroxyethyl)phenyl sulfate |
| Hydroxybenzaledhyde-O-glucuronide | (2S,3S,4S,5R)-6-(2-formyl-6-hydroxyphenyl)-3,4,5,6-tetrahydroxytetrahydro-2H-pyran-2-carboxylic acid |
| Benzoic acid-O-glucuronide | (2S,3S,4S,5R)-6-(2-carboxyphenyl)-3,4,5,6-tetrahydroxytetrahydro-2H-pyran-2-carboxylic acid |
| Vanillin-O-glucuronide | (2S,3S,4S,5R)-6-(4-formyl-2-methoxyphenoxy)-3,4,5-trihydroxytetrahydro-2H-pyran-2-carboxylic acid |
| Hydroxyphenylacetic acid-O-glucuronide | (2S,3S,4S,5R)-6-(carboxy(phenyl)methoxy)-3,4,5-trihydroxytetrahydro-2H-pyran-2-carboxylic acid |
| Coumaric acid-O-glucuronide | (2S,3S,4S,5R)-6-(4-((E)-2-carboxyvinyl)phenoxy)-3,4,5-trihydroxytetrahydro-2H-pyran-2-carboxylic acid |
| (iso)vanillic acid-O-glucuronide | (2S,3S,4S,5R)-6-(4-carboxy-2-methoxyphenoxy)-3,4,5-trihydroxytetrahydro-2H-pyran-2-carboxylic acid |
| Hippuric acid-O-glucuronide | (2S,3S,4S,5R)-6-(2-((carboxymethyl)carbamoyl)phenyl)-3,4,5,6-tetrahydroxytetrahydro-2H-pyran-2-carboxylic acid |
| Caffeic acid-O-glucuronide | (2S,3S,4S,5R)-6-(5-((E)-2-carboxyvinyl)-2-hydroxyphenoxy)-3,4,5-trihydroxytetrahydro-2H-pyran-2-carboxylic acid |
| Homovanillic acid-O-glucuronide | (2S,3S,4S,5R)-6-(4-(carboxymethyl)-2-methoxyphenoxy)-3,4,5-trihydroxytetrahydro-2H-pyran-2-carboxylic acid |
| Syringaldehyde-O-glucuronide | (2S,3S,4S,5R)-6-(4-formyl-2,6-dimethoxyphenoxy)-3,4,5-trihydroxytetrahydro-2H-pyran-2-carboxylic acid |
| Dihydroxycaffeic acid-O-glucuronide | (2S,3S,4S,5R)-6-(4-(2-carboxy-2,2-dihydroxyethyl)phenoxy)-3,4,5-trihydroxytetrahydro-2H-pyran-2-carboxylic acid |
| Dihydroferulic acid-O-glucuronide | (2S,3S,4S,5R)-6-(4-(2-carboxyethyl)-2-methoxyphenoxy)-3,4,5-trihydroxytetrahydro-2H-pyran-2-carboxylic acid |
| Syringic acid-O-glucuronide | (2S,3S,4S,5R)-6-(4-carboxy-2,6-dimethoxyphenoxy)-3,4,5-trihydroxytetrahydro-2H-pyran-2-carboxylic acid |

**Supporting Information Table 2 – sMRM transitions, parameters and detection limits of 30 identified oat bran-derived phenolics**

|  |  |  |  |  |  |  |  |
| --- | --- | --- | --- | --- | --- | --- | --- |
| **Metabolite** | **RT** | **MW** | **sMRM ion transitions (m/z)** | **Collision energy (V)** | **LOD (nM)** | **LOQ (nM)** | **R^2^** |
| *Aglycones* |  |  |  |  |  |  |  |
| 2,5-dihydroxybenzoic acid | 4.8 | 154.1 | 153 / 109 | -11 | 4.7 | 15.8 | 0.999 |
| 2,4-dihydroxybenzoic acid | 5.5 | 154.1 | 153 / 109 | -11 | 7.3 | 24.3 | 0.999 |
| Vanillic acid | 5.6 | 168.2 | 169 / 93 | 12 | 9.8 | 32.7 | 0.994 |
| Isovanillic acid | 6.0 | 168.2 | 169 / 93 | 12 | 7.3 | 24.4 | 0.999 |
| Syringic acid | 6.5 | 198.2 | 197 / 182 | -13 | 14.9 | 49.6 | 0.999 |
| Dihydroferulic acid | 7.1 | 196.2 | 195 / 136 | -15 | 21.5 | 71.8 | 1.000 |
| *p*-coumaric acid | 7.3 | 164.2 | 163 / 119 | -13 | 4.2 | 9.5 | 0.997 |
| Ferulic acid | 7.7 | 194.2 | 193 / 178 | -10 | 8.2 | 27.4 | 1.000 |
| Avenanthramide 2p | 9.9 | 299.3 | 298 / 254 | -17 | 0.8 | 2.7 | 0.994 |
| *Glycines* |  |  |  |  |  |  |  |
| 4-hydroxyhippuric acid | 4.4 | 195.2 | 194 / 100 | -11 | 4.3 | 14.2 | 0.997 |
| 3-hydroxyhippuric acid | 4.7 | 195.2 | 194 / 100 | -11 | 24.5 | 81.7 | 0.999 |
| 2-hydroxyhippuric acid | 7.0 | 195.2 | 194 / 100 | -11 | 2.9 | 9.7 | 0.998 |
| Feruloylglycine | 6.4 | 251.2 | 250 / 100*, 206, 191, 177, 149 | -11 | 2.2 | 7.2 | no standard |
| *Glucuronides* |  |  |  |  |  |  |  |
| Syringaldehyde OR Homovanillic acid OR  3,4-dihydroxyhydrocinamic acid-*O*-glucuronide^a,b)^ | 4.2 | 358.2 | 357 / 113* , 59 , 137 , 175 , 181 | -17, -23, -23, -11, -17 | 20.7 | 68.9 | no standard |
| Benzoic acid-*O*-glucuronide^a)^ | 5.1 | 314 | 313 / 113* , 175 , 137 , 93 | -17, -17, -17, -23 | 15.7 | 52.2 | no standard |
| Ferulic Acid-4-*O*-Glucuronide | 5.3 | 370.3 | 369 / 193* , 178 , 113 , 175 | -19, -23, -19, -10 | 21.4 | 71.3 | 0.997 |
| Vanillin OR 4-hydroxyphenylacetic acid-*O*-glucuronide^a,b)^ | 5.3 | 328.1 | 327 / 113* , 107 , 175 , 151 | -17, -23, -11, -11 | 35.0 | 116.7 | no standard |
| Dihydroferulic acid-4-*O*-glucuronide | 5.8 | 372.3 | 371 / 113* , 195 , 85 , 175 | -17, -20, -23, -17 | 11.7 | 39.1 | 0.990 |
| Isoferulic acid-*O*-glucuronide^a)^ | 6.1 | 370 | 369 / 193* , 178 , 113 , 175 | -19, -23, -19, -10 | 11.2 | 37.5 | no standard |
| Dihydroferulic acid-*O*-glucuronide^a)^ | 6.2 | 372.3 | 371 / 113* , 195 , 85 , 175 | -17, -20, -23, -17 | 8.0 | 26.5 | no standard |
| *Sulfates* |  |  |  |  |  |  |  |
| Dihydroxybenzoic acid-*O*-sulfate^a)^ | 3.0 | 234 | 233 / 109* , 189, 153, 97 | -23, -11, -23, -23 | 3.6 | 12.1 | no standard |
| Benzoic acid-*O*-sulfate^a)^ | 3.3 | 218 | 217 / 137*, 173 | -11, -11 | 5.7 | 19.3 | no standard |
| Syringaldehyde-*O*-sulfate^a)^ | 4.1 | 262.2 | 261 / 166* , 123 , 181 | -23, -23, -11 | 3.1 | 10.2 | no standard |
| Syringic acid-*O*-sulfate^a)^ | 4.1 | 278.2 | 277 / 197* , 182 , 153 , 123 | -11, -23, -23, -23 | 2.6 | 8.8 | no standard |
| Caffeic acid-*O*-sulfate^a)^ | 5.4 | 260.2 | 259 / 179* , 135 , 107 | -17, -23, -23 | 7.7 | 25.8 | no standard |
| Sinapic acid-*O*-sulfate^a)^ | 5.6 | 304.2 | 303 / 223* , 208 , 164 , 120 | -11, -23, -23, -23 | 1.6 | 5.3 | no standard |
| (Iso)ferulic acid-*O*-sulfate^a)^ | 5.7 | 274.2 | 273 / 178* , 192 , 134 | -20, -16, -25 | 1.6 | 5.3 | no standard |
| (Iso)ferulic acid-*O*-sulfate^a)^ | 5.9 | 274.2 | 273 / 178* , 192 , 134 | -20, -16, -25 | 1.5 | 5.0 | no standard |
| Isoferulic acid 3-*O*-sulfate | 6.1 | 274.2 | 273 / 178* , 192 , 134 | -20, -16, -25 | 1.4 | 4.7 | 1.000 |
| Vanillin or hydroxyphenylacetic acid-*O*-sulfate^a)^ | 6.4 | 232.1 | 231.1 / 151*, 136, 92 | -17, -23, -23 | 2.7 | 8.9 | no standard |
| *sMRM transition used for quantification; ^a)^ site of conjugation could not be ascertained as identification was based on known transitions where pure standards for isomers were not available or separation of isomers was chromatographically not possible; ^b)^tentatively identified compound which could have different isomeric configuration. Abbreviations: LOD, limit of detection (Signal/Noise = 3); LOQ, limit of quantification (Signal/Noise = 10); MW, molecular weight; RT, Retention time; sMRM, scheduled multiple reaction monitoring; R^2^, linear regression coefficient of standard curve; no standard, no analytical standard was available to make standard curve | | | | | | | |

# Supporting Information Table 3 – Urinary excretion rate per hour of oat bran-derived phenolic compounds after intake of 60g oat bran or a control meal in six healthy men (nmol/h)

|  |  |  |  |  |  |  |  |  |  |  |  |  |  |  |  |
| --- | --- | --- | --- | --- | --- | --- | --- | --- | --- | --- | --- | --- | --- | --- | --- |
|  |  |  |  |  |  |  |  |  |  |  |  |  | **Model P value** | | |
| **Metabolite** | **Meal** | **-12-0h** | **0-2h** | **2-4h** | **4-6h** | **6-8h** | **8-12h** | **12-24h** | **24-28h** | **28-32h** | **32-36h** | **36-48h** | **Inter-vention** | **Time** | **Inter-action** |
|  |  | µmol/h | | | | | | | | | | | | | |
| Avenanthramide A | Oats | 1 ± 0 | 3 ± 0**** | 1 ± 0 | 2 ± 0* | 2 ± 0 | 1 ± 0 | 1 ± 0 | 2 ± 0 | 1 ± 0 | 1 ± 0 | 1 ± 0 | *** | *** | *** |
|  | CON | 1 ± 0 | 1 ± 0 | 1 ± 0 | 1 ± 0 | 1 ± 0 | 1 ± 0 | 2 ± 0 | 1 ± 0 | 1 ± 0 | 1 ± 0 | 1 ± 0 |  |  |  |
| Ferulic acid | Oats | 9 ± 4 | 61 ± 15* | 29 ± 6 | 21 ± 3 | 41 ± 9 | 15 ± 5 | 15 ± 8 | 15 ± 3 | 24 ± 8 | 12 ± 4 | 13 ± 3 | 0.3 | **** | * |
|  | CON | 7 ± 1 | 21 ± 4 | 13 ± 4 | 18 ± 6 | 48 ± 23 | 25 ± 13 | 24 ± 13 | 14 ± 3 | 26 ± 5 | 23 ± 9 | 8 ± 3 |  |  |  |
| p-coumaric acid | Oats | 1 ± 0 | 7 ± 1** | 3 ± 0 | 3 ± 1 | 5 ± 0 | 2 ± 1 | 2 ± 0 | 2 ± 1 | 2 ± 0 | 2 ± 1 | 1 ± 0 | 0.1 | *** | * |
|  | CON | 2 ± 1 | 3 ± 1 | 2 ± 1 | 2 ± 0 | 3 ± 0 | 3 ± 2 | 3 ± 1 | 1 ± 0 | 2 ± 0 | 2 ± 1 | 1 ± 0 |  |  |  |
| Dihydroferulic acid | Oats | 34 ± 16 | 90 ± 42 | 34 ± 12 | 60 ± 17 | 378 ± 135**** | 109 ± 68 | 35 ± 17 | 23 ± 5 | 78 ± 22 | 60 ± 19 | 23 ± 6 | 0.1 | **** | *** |
|  | CON | 14 ± 5 | 56 ± 28 | 26 ± 8 | 26 ± 15 | 102 ± 38 | 38 ± 24 | 32 ± 10 | 24 ± 5 | 101 ± 66 | 84 ± 55 | 18 ± 5 |  |  |  |
| Isovanillic acid | Oats | 6 ± 5 | 24 ± 22 | 44 ± 5 | 69 ± 18 | 744 ± 470*** | 54 ± 51 | 22 ± 20 | 16 ± 3 | 188 ± 136 | 54 ± 44 | 31 ± 15 | 0.1 | * | * |
|  | CON | 7 ± 6 | 18 ± 17 | 30 ± NA | 62 ± 14 | 87 ± 39 | 4 ± 1 | 1 ± 0 | ND | 59 ± 17 | 4 ± 1 | 1 ± 0 |  |  |  |
| Syringic acid | Oats | 6 ± 2 | 29 ± 5 | 12 ± 3 | 18 ± 5 | 65 ± 20**** | 23 ± 10 | 6 ± 2 | 12 ± 2 | 14 ± 3 | 8 ± 4 | 6 ± 1 | * | **** | *** |
|  | CON | 2 ± 1 | 13 ± 3 | 12 ± 3 | 9 ± 1 | 16 ± 3 | 9 ± 2 | 8 ± 1 | 19 ± 7 | 14 ± 3 | 13 ± 4 | 4 ± 1 |  |  |  |
| 2,4-dihydroxybenzoic acid | Oats | 4 ± 1 | 15 ± 1 | 10 ± 3 | 54 ± 26* | 62 ± 18*** | 13 ± 5 | 23 ± 15 | 12 ± 2 | 43 ± 22 | 10 ± 3 | 8 ± 2 | ** | ** | *** |
|  | CON | 4 ± 1 | 11 ± 3 | 16 ± 6 | 6 ± 2 | 16 ± 5 | 6 ± 2 | 5 ± 2 | 18 ± 8 | 6 ± 2 | 6 ± 2 | 4 ± 2 |  |  |  |
| 2,5-dihydroxybenzoic acid | Oats | 51 ± 17 | 209 ± 58** | 78 ± 9 | 87 ± 15 | 97 ± 13 | 41 ± 11 | 27 ± 9 | 32 ± 10 | 28 ± 6 | 17 ± 6 | 27 ± 11 | 0.3 | **** | * |
|  | CON | 47 ± 9 | 88 ± 23 | 70 ± 24 | 63 ± 21 | 73 ± 15 | 34 ± 11 | 29 ± 16 | 40 ± 24 | 34 ± 19 | 43 ± 31 | 20 ± 8 |  |  |  |
| Vanillic acid | Oats | 29 ± 14 | 175 ± 36 | 80 ± 8 | 3352 ± 743** | 2918 ± 624* | 201 ± 98 | 58 ± 22 | 39 ± 12 | 1600 ± 600 | 168 ± 71 | 56 ± 21 | * | **** | * |
|  | CON | 13 ± 8 | 13 ± 7 | 41 ± 14 | 1281 ± 624 | 1594 ± 702 | 45 ± 33 | 15 ± 11 | 28 ± 5 | 1162 ± 513 | 128 ± 37 | 30 ± 20 |  |  |  |
| 2-hydroxyhippuric acid | Oats | 111 ± 35 | 371 ± 173* | 133 ± 35 | 294 ± 112 | 525 ± 82*** | 228 ± 75 | 137 ± 28 | 198 ± 107 | 133 ± 37 | 81 ± 21 | 117 ± 27 | ** | **** | ** |
|  | CON | 101 ± 21 | 113 ± 29 | 121 ± 32 | 84 ± 17 | 195 ± 20 | 101 ± 21 | 70 ± 24 | 54 ± 20 | 99 ± 41 | 158 ± 55 | 58 ± 20 |  |  |  |
| 3-hydroxyhippuric acid | Oats | 443 ± 148 | 2068 ± 536** | 958 ± 278 | 1142 ± 409 | 1643 ± 302 | 414 ± 124 | 452 ± 98 | 599 ± 209 | 650 ± 329 | 288 ± 75 | 557 ± 98 | * | **** | ** |
|  | CON | 395 ± 89 | 617 ± 203 | 729 ± 226 | 405 ± 140 | 981 ± 271 | 364 ± 97 | 541 ± 298 | 344 ± 223 | 297 ± 111 | 820 ± 325 | 355 ± 126 |  |  |  |
| 4-hydroxyhippuric acid | Oats | 553 ± 133 | 2262 ± 551** | 1289 ± 289 | 1566 ± 457 | 2479 ± 79 | 939 ± 348 | 748 ± 187 | 1108 ± 274 | 1302 ± 298 | 885 ± 228 | 899 ± 105 | 0.1 | **** | ** |
|  | CON | 430 ± 71 | 809 ± 232 | 1133 ± 342 | 807 ± 195 | 1875 ± 246 | 820 ± 105 | 628 ± 221 | 650 ± 247 | 1020 ± 334 | 1314 ± 307 | 528 ± 167 |  |  |  |
| Feruloylglycine | Oats | 11 ± 5 | 39 ± 12 | 11 ± 2 | 28 ± 8 | 56 ± 18** | 22 ± 10 | 13 ± 3 | 8 ± 2 | 15 ± 5 | 11 ± 4 | 12 ± 3 | 0.1 | **** | *** |
|  | CON | 9 ± 3 | 12 ± 3 | 9 ± 3 | 8 ± 1 | 24 ± 6 | 14 ± 3 | 14 ± 4 | 9 ± 3 | 19 ± 5 | 28 ± 10 | 8 ± 2 |  |  |  |
| Caffeic acid-sulfate | Oats | 68 ± 31 | 298 ± 127*** | 90 ± 38 | 128 ± 41 | 103 ± 25 | 23 ± 10 | 18 ± 7 | 13 ± 4 | 34 ± 15 | 17 ± 5 | 12 ± 4 | 0.6 | 0.1 | *** |
|  | CON | 65 ± 27 | 28 ± 8 | 15 ± 5 | 30 ± 12 | 137 ± 98 | 110 ± 84 | 47 ± 19 | 18 ± 6 | 76 ± 51 | 103 ± 62 | 22 ± 9 |  |  |  |
| (Iso)ferulic acid-*O*-sulfate | Oats | 78 ± 25 | 879 ± 142**** | 560 ± 104 | 474 ± 57 | 661 ± 119 | 245 ± 82 | 101 ± 36 | 170 ± 39 | 359 ± 76 | 219 ± 71 | 118 ± 29 | **** | **** | **** |
|  | CON | 48 ± 14 | 71 ± 20 | 282 ± 63 | 210 ± 51 | 454 ± 87 | 135 ± 31 | 79 ± 22 | 121 ± 54 | 235 ± 74 | 131 ± 58 | 70 ± 25 |  |  |  |
| (Iso)ferulic acid-*O*-sulfate | Oats | 6 ± 1 | 43 ± 7**** | 29 ± 8** | 20 ± 4 | 28 ± 6 | 12 ± 5 | 8 ± 1 | 8 ± 1 | 16 ± 3 | 10 ± 3 | 7 ± 1 | ** | **** | **** |
|  | CON | 5 ± 2 | 5 ± 1 | 13 ± 3 | 9 ± 1 | 25 ± 4 | 9 ± 2 | 6 ± 1 | 7 ± 2 | 15 ± 4 | 14 ± 4 | 5 ± 1 |  |  |  |
| Isoferulic acid-3-*O*-sulfate | Oats | 2 ± 0 | 23 ± 3**** | 16 ± 5 | 12 ± 1 | 7 ± 1 | 11 ± 6 | 3 ± 1 | 1 ± 0 | 4 ± 1 | 4 ± 2 | 3 ± 2 | 0.1 | *** | **** |
|  | CON | 1 ± 0 | 3 ± 1 | 9 ± 4 | 6 ± 2 | 12 ± 7 | 4 ± 2 | 2 ± 1 | 6 ± 3 | 7 ± 4 | 6 ± 3 | 3 ± 1 |  |  |  |
| Syringic acid-*O*-sulfate | Oats | 19 ± 8 | 99 ± 20*** | 54 ± 9 | 51 ± 10 | 165 ± 54**** | 76 ± 29 | 33 ± 11 | 41 ± 8 | 47 ± 9 | 32 ± 7 | 23 ± 4 | ** | **** | **** |
|  | CON | 15 ± 6 | 22 ± 9 | 58 ± 15 | 20 ± 4 | 68 ± 16 | 26 ± 9 | 22 ± 8 | 35 ± 12 | 51 ± 16 | 52 ± 16 | 13 ± 3 |  |  |  |
| Sinapic acid-*O*-sulfate | Oats | 40 ± 15 | 249 ± 55**** | 108 ± 14 | 144 ± 21 | 252 ± 75** | 66 ± 23 | 51 ± 17 | 55 ± 13 | 103 ± 20 | 49 ± 11 | 34 ± 6 | ** | **** | **** |
|  | CON | 28 ± 15 | 31 ± 13 | 59 ± 12 | 41 ± 8 | 130 ± 17 | 57 ± 25 | 45 ± 15 | 40 ± 11 | 101 ± 33 | 78 ± 27 | 32 ± 10 |  |  |  |
| Dihydroxybenzoic acid-*O*-sulfate | Oats | 24 ± 6 | 53 ± 13* | 27 ± 7 | 51 ± 12*** | 35 ± 7 | 16 ± 4 | 22 ± 6 | 16 ± 1 | 18 ± 4 | 18 ± 5 | 25 ± 4 | 0.2 | 0.1 | **** |
|  | CON | 16 ± 5 | 18 ± 2 | 14 ± 5 | 9 ± 2 | 28 ± 6 | 23 ± 8 | 26 ± 8 | 11 ± 2 | 18 ± 6 | 37 ± 18 | 17 ± 6 |  |  |  |
| Benzoic acid-*O*-sulfate | Oats | 596 ± 209 | 1697 ± 494 | 720 ± 219 | 1230 ± 451 | 1283 ± 274 | 1062 ± 359 | 863 ± 351 | 1034 ± 274 | 1125 ± 368 | 580 ± 236 | 852 ± 270 | 0.5 | * | * |
|  | CON | 506 ± 194 | 842 ± 251 | 935 ± 446 | 668 ± 213 | 988 ± 207 | 707 ± 162 | 819 ± 149 | 1073 ± 295 | 1146 ± 444 | 1066 ± 464 | 461 ± 143 |  |  |  |
| Vanillin or hydroxyphenylacetic acid-*O*-sulfate | Oats | 13 ± 9 | 81 ± 50*** | 22 ± 12 | 40 ± 23 | 29 ± 15 | 12 ± 7 | 14 ± 6 | 20 ± 11 | 13 ± 6 | 9 ± 3 | 9 ± 3 | 0.3 | * | * |
|  | CON | 4 ± 1 | 11 ± 7 | 6 ± 3 | 6 ± 3 | 9 ± 4 | 6 ± 3 | 7 ± 3 | 9 ± 5 | 7 ± 3 | 6 ± 2 | 7 ± 4 |  |  |  |
| Syringaldehyde OR homovanillic acid OR dihydroxyhydrocinamic acid sulfate | Oats | 13 ± 3 | 38 ± 9* | 21 ± 6 | 33 ± 9 | 46 ± 10 | 21 ± 8 | 15 ± 4 | 19 ± 2 | 32 ± 6 | 19 ± 4 | 14 ± 2 | 0.1 | **** | * |
|  | CON | 12 ± 3 | 17 ± 3 | 29 ± 8 | 20 ± 3 | 40 ± 11 | 15 ± 2 | 13 ± 2 | 18 ± 4 | 32 ± 9 | 27 ± 9 | 9 ± 1 |  |  |  |
| Ferulic Acid-4-*O*-glucuronide | Oats | 49 ± 20 | 265 ± 65**** | 205 ± 36 | 170 ± 27 | 214 ± 33 | 62 ± 23 | 61 ± 22 | 57 ± 14 | 128 ± 30 | 80 ± 28 | 52 ± 11 | **** | **** | **** |
|  | CON | 45 ± 12 | 44 ± 10 | 85 ± 20 | 67 ± 12 | 171 ± 21 | 75 ± 19 | 58 ± 9 | 45 ± 16 | 123 ± 25 | 101 ± 40 | 38 ± 8 |  |  |  |
| Isoferulic acid-*O*-glucuronide | Oats | 22 ± 6 | 142 ± 34**** | 94 ± 29 | 78 ± 14 | 104 ± 23 | 36 ± 32 | 23 ± 5 | 29 ± 7 | 58 ± 11 | 43 ± 15 | 25 ± 4 | * | **** | *** |
|  | CON | 17 ± 4 | 21 ± 5 | 39 ± 9 | 29 ± 5 | 67 ± 8 | 33 ± 6 | 20 ± 3 | 20 ± 6 | 57 ± 7 | 41 ± 9 | 15 ± 4 |  |  |  |
| Dihydroferulic acid-4-*O*-glucuronide | Oats | 3 ± 1 | 16 ± 5 | 11 ± 3 | 16 ± 5 | 49 ± 22**** | 27 ± 11 | 8 ± 4 | 8 ± 3 | 10 ± 2 | 10 ± 3 | 8 ± 3 | ** | *** | * |
|  | CON | 3 ± 1 | 6 ± 3 | 6 ± 1 | 8 ± 3 | 18 ± 6 | 7 ± 4 | 5 ± 2 | 5 ± 1 | 12 ± 7 | 13 ± 8 | 7 ± 2 |  |  |  |
| Dihydro(iso)ferulic acid-*O*-glucuronide | Oats | 31 ± 10 | 64 ± 16 | 21 ± 5 | 73 ± 20 | 189 ± 64**** | 69 ± 30 | 42 ± 12 | 29 ± 8 | 45 ± 10 | 53 ± 13 | 36 ± 9 | * | **** | ** |
|  | CON | 27 ± 8 | 29 ± 6 | 14 ± 3 | 17 ± 5 | 68 ± 25 | 46 ± 15 | 35 ± 7 | 21 ± 4 | 39 ± 18 | 92 ± 44 | 27 ± 12 |  |  |  |
| Benzoic acid-*O*-glucuornide | Oats | 23 ± 8 | 83 ± 26 | 28 ± 5 | 45 ± 15 | 78 ± 15** | 36 ± 11 | 34 ± 7 | 37 ± 14 | 29 ± 6 | 19 ± 4 | 23 ± 5 | *** | **** | * |
|  | CON | 27 ± 8 | 43 ± 10 | 31 ± 7 | 22 ± 4 | 36 ± 5 | 19 ± 3 | 23 ± 5 | 24 ± 9 | 25 ± 3 | 30 ± 12 | 19 ± 9 |  |  |  |
| Syringaldehyde OR Homovanillic acid OR 3,4-dihydroxyhydrocinamic acid-*O*-glucuronide | Oats | 128 ± 30 | 528 ± 146** | 205 ± 37 | 176 ± 53 | 273 ± 67 | 155 ± 31 | 167 ± 30 | 267 ± 60 | 142 ± 40 | 179 ± 47 | 203 ± 29 | 0.8 | *** | ** |
|  | CON | 68 ± 27 | 201 ± 66 | 208 ± 61 | 95 ± 15 | 203 ± 40 | 141 ± 37 | 177 ± 34 | 179 ± 37 | 131 ± 32 | 211 ± 46 | 131 ± 32 |  |  |  |
| Vanillin OR 4-hydroxyphenylacetic acid-*O*-glucuronide | Oats | 14 ± 7 | 39 ± 16 | 48 ± 18 | 134 ± 36**** | 212 ± 27**** | 60 ± 22 | 26 ± 8 | 19 ± 3 | 20 ± 6 | 16 ± 3 | 14 ± 3 | *** | **** | **** |
|  | CON | 10 ± 6 | 15 ± 7 | 22 ± 20 | 15 ± 5 | 36 ± 8 | 15 ± 2 | 17 ± 2 | 12 ± 2 | 19 ± 3 | 20 ± 4 | 11 ± 4 |  |  |  |
| Data are reported as mean ± SEM and were analysed by two-factor repeated measurement linear mixed model with time and treatment as the two factors. When the interaction effect was significant, post-hoc analyses with Tukey-Kramer adjustment were performed. If excretion was ND, the concentration was replaced with the limit of detection for statistical analysis. Model P values in the three far right columns and post-hoc P values next to the excretion rates are indicated as follow: * P < 0.05; ** P <0.01; *** P<0.001; **** P<0.0001. ND; not detected; CON control. | | | | | | | | | | | | | | | |

**Supporting Information Table 4 – sMRM transitions, parameters and detection limits of non oat bran-derived phenolics**

|  |  |  |  |  |  |  |  |
| --- | --- | --- | --- | --- | --- | --- | --- |
| **Metabolite** | **RT** | **MW** | **sMRM ion transitions (m/z)** | **Collision energy (V)** | **LOD (nM)** | **LOQ (nM)** | **R^2^** |
| 3,4-dihydrocaffeic acid | 6.4 | 182.17 | 181.17 / 137 | -10 | 17.9 | 59.7 | 0.993 |
| 4-hydroxybenzaldehyde | 5.9 | 122.12 | 121.12 / 92 | -20 | 31.7 | 105.8 | 0.995 |
| 4-hydroxyphenylacetic acid | 6.5 | 152.15 | 151.14 / 107 | -10 | 56.3 | 187.8 | 0.997 |
| 3-hydroxyphenylacetic acid | 6.3 | 152.15 | 151.14 / 107 | -10 | 64.8 | 215.9 | 0.996 |
| Dihydroisoferulic acid | 7.5 | 196.2 | 195 / 136 | -15 | 35.2 | 117.5 | 1.000 |
| Isoferulic acid | 8.0 | 195.2 | 194.2 / 136 | -15 | 16.1 | 53.8 | 1.000 |
| o-coumaric acid | 8.4 | 164.2 | 163 / 119 | -13 | 44.8 | 149.4 | 0.997 |
| Salicylic acid | 8.1 | 138.1 | 137.12 / 93 | -23 | 41.0 | 136.6 | 0.999 |
| Syringaldehyde | 7.5 | 182.2 | 181.17 / 151 | -18 | 0.3 | 1.0 | 0.993 |
| Vanillin | 6.9 | 152.1 | 151.15 / 136 | -12 | 42.8 | 142.8 | 0.999 |
| 4-hydroxybenzoic acid | 4.9 | 138.1 | 137.12 / 93 | -13 | 64.8 | 216.1 | 0.998 |
| Caffeic acid | 6.0 | 180.2 | 179.16 / 135 | -15 | 124.1 | 413.7 | 0.988 |
| Gallic acid | 1.7 | 170.1 | 169.12 / 125 | -13 | 6.5 | 21.7 | 0.998 |
| Hippuric acid | 5.7 | 179.2 | 178.17 / 134 | -11 | 211.6 | 705.5 | 0.995 |
| Homovanillic acid | 6.2 | 182.2 | 181.17 / 137 | -10 | 44.4 | 148.0 | 0.999 |
| Protocatechuic acid OR 3,5-dihydroxybenzoic acid^a)^ | 3.7 | 154.1 | 153.12 / 109 | -13 | 64.1 | 213.7 | 0.998 |
| Salicylic acid-*O*-sulfate ^a,b)^ | 3.2 | 218.1 | 217 / 137* , 93 , 79 | -11, -23, -17 | 75.5 | 251.6 | no standard |
| Dihydroxybenzoic acid-*O*-sulfate ^a,b)^ | 2.7 | 234.0 | 233 / 153* , 189 , 109 | -23, -11, -23 | 76.8 | 255.9 | no standard |
| Coumaric acid-*O*-sulfate ^a,b)^ | 9.0 | 244.2 | 243 / 163* , 93 , 79 | -17, -23, -23 | 38.0 | 126.6 | no standard |
| (iso)vanillic acid-*O*-sulfate ^a,b)^ | 4.2 | 248.0 | 247 / 167* , 152 , 123, 108 | -23, -17, -17, -23 | 80.4 | 267.9 | no standard |
| Hippuric acid-*O*-sulfate ^a,b)^ | 3.4 | 259.2 | 258 / 178* , 79 , 134 | -17, -23, -23 | 77.2 | 257.3 | no standard |
| Hippuric acid-*O*-sulfate ^a,b)^ | 3.1 | 259.2 | 258 / 178* , 79 , 134 | -17, -23, -23 | 59.4 | 198.1 | no standard |
| Hippuric acid-*O*-sulfate ^a,b)^ | 3.8 | 259.2 | 258 / 178* , 79 , 134 | -17, -23, -23 | 38.4 | 127.9 | no standard |
| Homovanillic acid OR dihydrocaffeic acid-*O*-sulfate ^a,b)^ | 4.6 | 262.2 | 261 / 181* , 121 , 79, 137 | -17, -23, -23, -23 | 121.1 | 403.6 | no standard |
| Homovanillic acid OR dihydrocaffeic-*O*-sulfate ^a,b)^ | 4.9 | 262.2 | 261 / 181* , 121 , 79, 137 | -17, -23, -23, -23 | 155.5 | 518.2 | no standard |
| Hydroxybenzaledhyde-*O*-glucuronide ^a,b)^ | 8.3 | 298.1 | 297 / 113* , 175 , 121 | -11, -11, -23 | 50.8 | 169.2 | no standard |
| Benzoic acid-*O*-glucuronide ^a,b)^ | 2.7 | 314.0 | 313 / 137* , 175 , 113, 93 | -17, -11, -11, -23 | 48.9 | 162.9 | no standard |
| Benzoic acid-*O*-glucuronide ^a,b)^ | 3.8 | 314.0 | 313 / 137* , 175 , 113, 93 | -17, -11, -11, -23 | 55.9 | 186.3 | no standard |
| Benzoic acid-*O*-glucuronide ^a,b)^ | 4.6 | 314.0 | 313 / 137* , 175 , 113, 93 | -17, -11, -11, -23 | 93.7 | 312.5 | no standard |
| Benzoic acid-*O*-glucuronide ^a,b)^ | 4.8 | 314.0 | 313 / 137* , 175 , 113, 93 | -17, -11, -11, -23 | 44.9 | 149.6 | no standard |
| Salicylic acid-*O*-glucuronide ^a,b)^ | 7.8 | 314.1 | 313 / 137* , 93 , 75, 175, 113 | -11, -23, -11, -11, -11 | 41.2 | 137.2 | no standard |
| Salicylic acid-*O*-glucuronide ^a,b)^ | 7.7 | 314.1 | 313 / 137* , 93 , 75, 175, 113 | -11, -23, -11, -11, -11 | 46.5 | 155.0 | no standard |
| Vanillin OR hydroxyphenylacetic acid-*O*-glucuronide ^a,b)^ | 4.5 | 328.1 | 327 / 113* , 136 , 175, 151 | -11, -23, -11, -23 | 111.6 | 372.1 | no standard |
| Coumaric acid-*O*-glucuronide ^a,b)^ | 9.1 | 328.1 | 327 / 113* , 107 , 175, 151 | -17, -23, -11, -11 | 131.5 | 438.3 | no standard |
| Coumaric acid-*O*-glucuronide ^a,b)^ | 10.3 | 340.2 | 339 / 113* , 117 , 175, 163 | -11, -23, -11, -17 | 62.9 | 209.7 | no standard |
| (iso)vanillic acid-*O*-glucuronide ^a,b)^ | 4.0 | 344.0 | 339 / 113* , 117 , 175, 163 | -11, -23, -11, -17 | 85.4 | 284.6 | no standard |
| (iso)vanillic acid-*O*-glucuronide ^a,b)^ | 4.5 | 344.0 | 343 / 113* , 175 , 167, 152, 108 | -17, -17, -17, -23, -23 | 102.6 | 341.9 | no standard |
| Hippuric acid-*O*-glucuronide ^a,b)^ | 4.9 | 355.2 | 354 / 113* , 134 , 175, 178 | -11, -23, -11, -17 | 56.5 | 188.3 | no standard |
| Caffeic acid-*O*-glucuronide ^a,b)^ | 6.1 | 356.2 | 355 / 113* , 135 , 175, 179 | -17, -23, -11, -17 | 109.2 | 364.2 | no standard |
| Syringaldehyde OR Homovanillic acid OR dihydrocaffeic-*O*-glucuronide ^a,b)^ | 5.1 | 358.2 | 357 / 113* , 175 , 181, 166, 123 | -11, -11, -17, -23, -23 | 84.1 | 280.2 | no standard |
| Syringaldehyde OR Homovanillic acid OR dihydroxycaffeic acid-*O*-glucuronide ^a,b)^ | 3.9 | 358.2 | 357 / 113* , 175 , 181, 166, 123 | -11, -11, -17, -23, -23 | 70.5 | 235.0 | no standard |
| Dihydroferulic acid-*O*-glucuronide ^a,b)^ | 5.1 | 372.3 | 371 / 113* , 195 , 85, 175 | -17, -20, -23, -17 | 78.1 | 260.5 | 0.990 |
| Syringic acid-*O*-glucuronide ^a,b)^ | 7.5 | 374.2 | 373 / 113* , 175 , 197, 153 | -17, -11, -11, -17 | 74.8 | 249.4 | no standard |
| Syringic acid-*O*-glucuronide ^a,b)^ | 9.0 | 374.2 | 373 / 113* , 175 , 197, 153 | -17, -11, -11, -17 | 77.6 | 258.6 | no standard |
| *sMRM transition used for quantification; ^a)^ site of conjugation could not be ascertained as identification was based on known transitions where pure standards for isomers were not available or separation of isomers was chromatographically not possible; ^b)^tentatively identified compound which could have different isomeric configuration. Abbreviations: LOD, limit of detection (Signal/Noise = 3); LOQ, limit of quantification (Signal/Noise = 10); MW, molecular weight; RT, Retention time; sMRM, scheduled multiple reaction monitoring; R^2^, linear regression coefficient of standard curve; no standard, no analytical standard was available to make standard curve | | | | | | | |

# Supporting Information Table 5 – Urinary excretion rate per hour of non oat bran-derived phenolic compounds after intake of 60g oat bran or a control meal in six healthy men (nmol/h)

|  |  |  |  |  |  |  |  |  |  |  |  |  |  |  |  |  |
| --- | --- | --- | --- | --- | --- | --- | --- | --- | --- | --- | --- | --- | --- | --- | --- | --- |
| **Non-metabolite** | **RT**  **(min)** | **Meal** | **-12-0h** | **0-2h** | **2-4h** | **4-6h** | **6-8h** | **8-12h** | **12-24h** | **24-28h** | **28-32h** | **32-36h** | **36-48h** | **Inter-vention** | **Time** | **Inter-action** |
|  |  |  | (nmol/h) | | | | | | | | | | |  |  |  |
| 3,4-dihydrocaffeic acid | 6.4 | Oats | 8±5 | 11±2 | ND | 54±11 | 164±31 | 60±28 | 15±4 | 6±1 | 81±20 | 63±26 | 18±5 | 0.2 | **** | 0.7 |
|  |  | CON | 10±3 | 10±4 | 9±4 | 50±11 | 138±23 | 47±16 | 20±5 | 6±3 | 100±17 | 44±13 | 13±4 |  |  |  |
| 4-hydroxybenzaldehyde | 5.9 | Oats | 9±3 | 43±12 | 29±6 | 37±11 | 48±9 | 23±8 | 8±3 | 24±7 | 30±9 | 18±6 | 12±3 | 0.5 | **** | 0.2 |
|  |  | CON | 4±2 | 11±4 | 24±9 | 17±6 | 35±6 | 16±5 | 12±5 | 15±7 | 30±10 | 21±5 | 8±3 |  |  |  |
| 4-hydroxyphenylacetic acid | 6.5 | Oats | 2944±997 | 7303±888 | 3598±538 | 4737±1289 | 9307±1394 | 3870±1227 | 2667±686 | 3229±754 | 4045±1013 | 3609±1237 | 3379±533 | 0.2 | **** | 0.5 |
|  |  | CON | 2658±622 | 5407±884 | 5961±2151 | 4948±859 | 9741±1885 | 4846±1390 | 3459±1119 | 4297±1125 | 4778±1278 | 6608±3173 | 2256±698 |  |  |  |
| 3-hydroxyphenylacetic acid | 6.3 | Oats | 21867±6765 | 63086±16587 | 29542±6008 | 39413±11293 | 72562±8932 | 26418±7768 | 23593±7204 | 39232±10190 | 39862±8965 | 26007±8449 | 30693±5066 | 0.3 | **** | 0.1 |
|  |  | CON | 20461±4935 | 44887±8620 | 47366±13805 | 27549±6555 | 63817±5442 | 23306±2171 | 27629±7689 | 25976±8532 | 33403±10462 | 38274±13067 | 15391±4559 |  |  |  |
| Dihydroisoferulic acid | 7.5 | Oats | 15±7 | 36±23 | 23±8 | 22±11 | 42±4 | 7±3 | 20±5 | 28±18 | 25±12 | 16±7 | 19±6 | 0.2 | * | 0.6 |
|  |  | CON | 7±3 | 10±3 | 18±16 | 53± | 27±18 | 7±2 | 9±2 | 18±7 | 16±6 | 13±7 | 6±2 |  |  |  |
| Isoferulic acid | 8.0 | Oats | 17±5 | 99±59 | 96±29 | 58±12 | 148±46 | 22± | 7±1 | 13±7 | 61±20 | 47±22 | 9±2 | 0.2 | *** | 0.5 |
|  |  | CON | 15±3 | 20±7 | 24±16 | 24± | 130±22 | 41±12 | 20±1 | 23±1 | 41±19 | 36±12 | 6± |  |  |  |
| o-coumaric acid | 8.4 | Oats | 2± | ND | ND | 11± | 17±10 | ND | 1±0 | 1±0 | 26±14 | 3± | 0± | 0.6 | * | 0.4 |
|  |  | CON | 0± | 4±3 | 3±3 | 9±6 | 39±3 | 5± | 1±0 | 1± | 22±9 | 2±1 | 0± |  |  |  |
| Salicylic acid | 8.1 | Oats | 67±59 | 171±64 | 143±54 | 77±21 | 222±120 | 39±19 | 17±5 | 95±66 | 103±36 | 109±82 | 30±14 | ** | 0.1 | 0.2 |
|  |  | CON | 6±2 | 17±7 | 39±17 | 32±12 | 43±7 | 24±11 | 11±5 | 20±9 | 27±8 | 24±8 | 9±3 |  |  |  |
| Syringaldehyde | 7.5 | Oats | 0±0 | 0±0 | 0±0 | 1±0 | 0±0 | ND | 0± | 0± | 0±0 | ND | 0±0 | 0.2 | ** | 0.4 |
|  |  | CON | 0±0 | 0±0 | 0± | 0± | 0±0 | 0±0 | 0±0 | 0± | 0± | 1± | 0±0 |  |  |  |
| Vanillin | 6.9 | Oats | 3±1 | 11±3 | 13±3 | 18±2 | 24±2 | 9±4 | 4±1 | 8±3 | 17±3 | 8±2 | 4±1 | 0.2 | **** | 0.3 |
|  |  | CON | 2±0 | 3±1 | 10±3 | 18±6 | 19±2 | 4±1 | 4±1 | 5±1 | 20±2 | 7±1 | 3±1 |  |  |  |
| 4-hydroxybenzoic acid | 4.9 | Oats | 75±22 | 337±83 | 182±40 | 224±31 | 340±41 | 164±50 | 146±67 | 208±45 | 226±56 | 147±44 | 185±58 | 0.5 | **** | 0.7 |
|  |  | CON | 53±10 | 256±123 | 211±86 | 204±99 | 343±118 | 182±64 | 208±113 | 165±64 | 162±55 | 171±49 | 85±42 |  |  |  |
| Caffeic acid | 6.0 | Oats | 34±8 | 119±29 | 91±26 | 111±19 | 161±18 | 80±35 | 53±13 | 73±18 | 81±18 | 62±20 | 62±9 | 0.1 | **** | 0.4 |
|  |  | CON | 30±4 | 61±16 | 87±24 | 67±15 | 128±21 | 60±7 | 47±15 | 46±18 | 78±30 | 80±23 | 33±10 |  |  |  |
| Gallic acid | 1.7 | Oats | 1±1 | 1±0 | 2±1 | 2±1 | 2±1 | 3±2 | 1±1 | 2±1 | 1±0 | 2±1 | 1±1 | 0.8 | 0.8 | 0.8 |
|  |  | CON | 0±0 | 1±1 | 2±2 | 2±1 | 1±0 | 0±0 | 0±0 | 1±0 | 1±0 | 5±4 | 0±0 |  |  |  |
| Hippuric acid | 5.7 | Oats | 2772±703 | 9454±2193 | 7027±2028 | 7653±2036 | 12773±1494 | 5060±2449 | 4158±1001 | 5857±1432 | 6817±1545 | 5006±1627 | 4930±683 | 0.1 | **** | 0.4 |
|  |  | CON | 2403±339 | 5052±1396 | 7131±1944 | 5439±1199 | 10688±1668 | 4905±592 | 3905±1209 | 3593±1437 | 6311±2403 | 6525±1869 | 2696±853 |  |  |  |
| Homovanillic acid | 6.2 | Oats | 251±64 | 894±261 | 584±76 | 726±164 | 1600±698 | 437±132 | 284±47 | 535±97 | 553±104 | 320±78 | 399±80 | 0.5 | **** | 0.8 |
|  |  | CON | 357±102 | 995±393 | 1116±189 | 1098±599 | 1616±309 | 657±316 | 352±81 | 959±384 | 1409±771 | 943±475 | 361±145 |  |  |  |
| Protocatechuic acid OR 3,5-dihydroxybenzoic acid | 3.7 | Oats | 8±2 | 24±5 | 11±2 | 14±5 | 22±3 | 10±3 | 12±4 | 16±3 | 21±6 | 14±4 | 13±2 | 0.7 | 0.5 | 0.1 |
|  |  | CON | 7±2 | 14±3 | 14±3 | 13±2 | 26±3 | 13±2 | 14±4 | 18±3 | 26±2 | 20±4 | 9±2 |  |  |  |
| Salicylic acid-*O*-sulfate | 3.2 | Oats | 574±202 | 1619±473 | 689±213 | 1180±435 | 1224±269 | 1005±343 | 828±336 | 999±263 | 1094±362 | 563±231 | 802±281 | 0.5 | * | * |
|  |  | CON | 488±190 | 804±242 | 903±434 | 640±208 | 944±207 | 675±156 | 775±147 | 1041±296 | 1107±435 | 1029±451 | 442±141 |  |  |  |
| Dihydroxybenzoic acid-*O*-sulfate | 2.7 | Oats | 11±4 | 19±7 | 5±1 | 15±8 | 14±3 | 7±2 | 19±7 | 8±1 | 10±3 | 6±1** | 14±4 | 0.1 | 0.3 | ** |
|  |  | CON | 9±2 | 13±3 | 9±4 | 7±2 | 18±5 | 21±8 | 14±3 | 8±2 | 13±4 | 26±13 | 7±2 |  |  |  |
| Coumaric acid-*O*-sulfate | 9.0 | Oats | 27±6 | 30±10 | 11±2 | 21±5 | 35±10 | 20±4 | 22±7 | 14±4 | 18±8 | 11±4 | 13±2 | 0.3 | 0.5 | 0.2 |
|  |  | CON | 8±1 | 17±7 | 17±6 | 13±2 | 26±4 | 128±110 | 11±2 | 18±6 | 29±18 | 70±34 | 59±39 |  |  |  |
| (iso)vanillic acid-*O*-sulfate | 4.2 | Oats | 27±9 | 153±35 | 61±13 | 2956±767 | 3392±823 | 245±90 | 65±25 | 36±4 | 2658±490 | 168±45 | 61±11 | 0.7 | **** | 0.9 |
|  |  | CON | 32±9 | 27±7 | 42±13 | 2623±448 | 3272±883 | 217±53 | 65±19 | 28±7 | 2979±684 | 240±65 | 70±22 |  |  |  |
| Hippuric acid-*O*-sulfate | 3.4 | Oats | 120±55 | 310±109 | 89±29 | 139±70 | 160±29 | 61±13 | 72±20 | 105±26 | 73±20 | 52±12 | 101±28 | 0.9 | *** | 0.1 |
|  |  | CON | 106±38 | 180±51 | 137±53 | 68±19 | 109±28 | 54±16 | 93±37 | 90±38 | 68±29 | 182±128 | 69±20 |  |  |  |
| Hippuric acid-*O*-sulfate | 3.1 | Oats | 4±1 | 9±3 | 3±1 | 5±2 | 6±1 | 3±1 | 6±3 | 5±1 | 4±2 | 3±1 | 5±2 | 0.4 | 0.5 | 0.6 |
|  |  | CON | 12±9 | 9±3 | 4±2 | 5±2 | 7±3 | 5±2 | 6±2 | 5±1 | 4±1 | 7±3 | 3±1 |  |  |  |
| Hippuric acid-*O*-sulfate | 3.8 | Oats | 91±44 | 216±94 | 147±76 | 171±78 | 213±93 | 179±98 | 143±64 | 174±70 | 215±94 | 86±55 | 183±89 | * | 0.1 | 0.3 |
|  |  | CON | 59±37 | 156±104 | 141±78 | 127±85 | 166±92 | 128±82 | 112±58 | 185±102 | 173±87 | 202±103 | 118±78 |  |  |  |
| Homovanillic acid OR dihydroxyhydroycinamic acid-*O*-sulfate | 4.6 | Oats | 168±19 | 350±68 | 113±22 | 179±49 | 260±40 | 161±35 | 184±52 | 236±18 | 212±39 | 162±25 | 206±37 | 0.3 | ** | 0.2 |
|  |  | CON | 158±35 | 278±38 | 218±63 | 154±25 | 281±40 | 185±28 | 231±48 | 199±29 | 216±60 | 322±163 | 128±27 |  |  |  |
| Homovanillic acid34dihydrocaffeic-*O*-sulfate | 4.9 | Oats | 115±35 | 152±49 | 32±9 | 117±29 | 171±45 | 172±88 | 88±25 | 60±11 | 101±31 | 191±81 | 172±20 | ** | *** | 0.3 |
|  |  | CON | 165±43 | 101±31 | 41±11 | 56±20 | 91±23 | 115±40 | 125±31 | 37±4 | 43±14 | 190±65 | 74±34 |  |  |  |
| Hydroxybenzaledhyde-*O*-glucuronide | 8.3 | Oats | 65±52 | 186±137 | 151±134 | 137±102 | 156±95 | 168±154 | 74±39 | 103±36 | 143±84 | 70±31 | 211±136 | 0.5 | 0.8 | 0.5 |
|  |  | CON | 48±33 | 116±78 | 194±166 | 151±79 | 104±71 | 68±37 | 104±44 | 127±36 | 142±31 | 145±79 | 56±13 |  |  |  |
| Benzoic acid-*O*-glucuronide | 2.7 | Oats | 11±3 | 35±10 | 12±5 | 18±8 | 18±4 | 12±3 | 23±10 | 21±9 | 19±7 | 13±4 | 17±6 | 0.3 | * | 0.5 |
|  |  | CON | 9±5 | 18±8 | 18±12 | 13±6 | 20±10 | 11±6 | 15±6 | 17±6 | 17±6 | 16±8 | 6±2 |  |  |  |
| Benzoic acid-*O*-glucuronide | 3.8 | Oats | 11±2 | 35±7 | 15±3 | 18±7 | 24±5 | 11±3 | 14±5 | 15±4 | 14±4 | 13±3 | 13±3 | 0.8 | **** | 0.1 |
|  |  | CON | 7±2 | 16±4 | 15±5 | 10±2 | 19±3 | 12±2 | 13±2 | 11±3 | 12±3 | 16±7 | 8±2 |  |  |  |
| Benzoic acid-*O*-glucuronide | 4.6 | Oats | 3±1 | 7±2 | 6±1 | 8±3 | 14±5 | 3±1 | 3±1 | 4±1 | 5±1 | 4±1 | 3±0 | 0.3 | **** | 0.3 |
|  |  | CON | 2±1 | 5±1 | 6±2 | ND | 7±1 | 3±1 | 3±0 | 3±0 | 4±1 | 5±2 | 3±1 |  |  |  |
| Benzoic acid-*O*-glucuronide | 4.8 | Oats | 2±1 | ND** | ND | ND | ND | ND | 1± | ND | ND | ND | 2±1 | 0.1 | **** | ** |
|  |  | CON | 2±0 | 2±1 | ND | ND | ND | 3±0 | 2±0 | 2±1 | ND | 6±2 | 1±1 |  |  |  |
| Salicylic acid-*O*-glucuronide | 7.8 | Oats | 2±1 | 5±1 | ND | 5±3 | 12± | 2±1 | 3±1 | 2±1 | 2± | 1± | 3±1 | 0.4 | *** | 0.8 |
|  |  | CON | 3±1 | 7±3 | 7±6 | 6±2 | 11±5 | 5±2 | 3±1 | 4±0 | 7±3 | 4±2 | 2±1 |  |  |  |
| Salicylic acid-*O*-glucuronide | 7.7 | Oats | 2±1 | 7±1 | ND | 11± | 5±2 | 2±1 | 3±2 | 3±1 | 3± | 1± | 3±2 | 0.9 | *** | 0.9 |
|  |  | CON | 3±2 | 8±4 | 13±13 | 7±3 | 14±10 | 5±2 | 3±1 | 4±1 | 5±1 | 5±2 | 2±1 |  |  |  |
| Vanillin OR hydroxyphenylacetic acid-*O*-glucuronide | 4.5 | Oats | 26±9 | 68±14 | 32±6 | 44±14 | 71±11 | 27±7 | 30±8 | 34±7 | 37±8 | 32±10 | 34±7 | 0.3 | *** | 0.2 |
|  |  | CON | 18±3 | 39±5 | 49±12 | 31±4 | 65±12 | 35±5 | 39±5 | 27±6 | 32±6 | 58±38 | 17±7 |  |  |  |
| Coumaric acid-*O*-glucuronide | 9.1 | Oats | 19±8 | 55±24 | 22±2 | 29±8 | 35±3 | 26±11 | 24±5 | 17±5 | 21±1 | 17±9 | 15±3 | * | 0.5 | 0.2 |
|  |  | CON | 14±4 | 37±18 | 33±17 | 24±7 | 29±4 | 96±70 | 46±32 | 23±6 | 36±23 | 120±80 | 60±43 |  |  |  |
| Coumaric acid-*O*-glucuronide | 10.3 | Oats | 43±32 | 58±21 | 17±5 | 21±9 | 23±6 | 8±3 | 11±3 | 10±4 | 8±2 | 11±4 | 15±3 | 1 | 0.2 | 0.5 |
|  |  | CON | 150±137 | 29±12 | 23±5 | 23±11 | 35±16 | 47±36 | 115±102 | 17±10 | 20±11 | 18±8 | 9±3 |  |  |  |
| (iso)vanillic acid-*O*-glucuronide | 4.0 | Oats | 35±12 | 127±38 | 93±12 | 1720±453 | 3518±501 | 672±248 | 143±38 | 44±8 | 1811±500 | 676±222 | 149±31 | * | **** | 0.9 |
|  |  | CON | 49±14 | 46±11 | 52±13 | 1466±284 | 3110±367 | 591±152 | 150±25 | 32±8 | 1628±449 | 528±114 | 163±62 |  |  |  |
| (iso)vanillic acid-*O*-glucuronide | 4.5 | Oats | 165±52 | 677±159 | 367±54 | 7116±1491 | 10045±1653 | 1591±596 | 382±118 | 214±30 | 6104±1656 | 1568±521 | 478±96 | 0.2 | **** | 1 |
|  |  | CON | 187±44 | 237±68 | 219±52 | 6227±994 | 9168±1313 | 1512±396 | 450±63 | 147±33 | 5892±1604 | 1459±320 | 424±130 |  |  |  |
| Hippuric acid-*O*-glucuronide | 4.9 | Oats | 54±21 | 144±48 | 58±20 | 84±38 | 103±25 | 47±20 | 50±22 | 65±24 | 84±30 | 64±22 | 50±19 | 0.8 | *** | 0.2 |
|  |  | CON | 31±14 | 54±27 | 71±43 | 38±20 | 96±40 | 44±20 | 38±14 | 48±18 | 63±22 | 56±30 | 30±14 |  |  |  |
| Caffeic acid-*O*-glucuronide | 6.1 | Oats | 16±7 | 57±17 | 19± | 21±3 | 25±9 | 5±2 | 8±2 | 13±3 | 7±0 | 12±4 | 23±2 | 0.7 | 0.2 | 0.1 |
|  |  | CON | 33±24 | 25±4 | 13±1 | 16±5 | 24±5 | 19±7 | 40±22 | 11±5 | 22±16 | 18±7 | 20±9 |  |  |  |
| Syringaldehyde OR Homovanillic acid OR dihydrocaffeic-*O*-glucuronide | 5.1 | Oats | 13±3 | 45±11 | 22±4 | 31±6 | 47±7 | 23±6 | 24±5 | 32±7 | 30±5 | 24±6 | 26±4 | 0.1 | **** | 0.4 |
|  |  | CON | 16±4 | 29±5 | 29±7 | 24±4 | 42±4 | 25±4 | 28±4 | 26±4 | 30±4 | 34±13 | 17±5 |  |  |  |
| Syringaldehyde OR Homovanillic acid OR dihydroxycaffeic acid-*O*-glucuronide | 3.9 | Oats | 7±1 | 21±2 | 12±2 | 14±3 | 21±3 | 12±4 | 9±2 | 12±2 | 14±3 | 11±3 | 9±2 | 0.1 | **** | 0.4 |
|  |  | CON | 5±1 | 11±3 | 13±3 | 10±2 | 17±1 | 9±2 | 8±1 | 8±2 | 13±2 | 12±3 | 6±1 |  |  |  |
| Dihydroferulic acid-*O*-glucuronide | 5.1 | Oats | 6±2 | 17±4 | 8±2 | 10±4 | 21±5 | 5±1 | 7±2 | 7±2 | 16±5 | 9±2 | 7±2 | 0.5 | *** | 0.4 |
|  |  | CON | 5±1 | 7±1 | 8±3 | 17±7 | 38±19 | 11±3 | 7±1 | 6±1 | 15±5 | 11±3 | 5±1 |  |  |  |
| Syringic acid-*O*-glucuronide | 7.5 | Oats | 249±66 | 700±75 | 268±41 | 399±60 | 535±77 | 276±79 | 357±55 | 407±72 | 327±80 | 319±101 | 415±56 | 0.5 | *** | 0.1 |
|  |  | CON | 258±38 | 426±44 | 333±67 | 276±28 | 498±46 | 294±25 | 390±37 | 316±49 | 400±73 | 479±157 | 258±65 |  |  |  |
| Syringic acid-*O*-glucuronide | 9.0 | Oats | 56±29 | 183±47 | 126±66 | 194±71 | 277±77 | 175±82 | 132±31 | 178±53 | 146±55 | 141±54 | 145±20 | * | **** | 0.4 |
|  |  | CON | 88±31 | 171±54 | 140±34 | 150±47 | 259±60 | 175±59 | 138±31 | 144±44 | 213±67 | 197±55 | 112±44 |  |  |  |
| Data are reported as mean ± SEM and were analysed by two-factor repeated measurement linear mixed model with time and treatment as the two factors. When the interaction effect was significant, post-hoc analyses with Tukey-Kramer adjustment were performed. If excretion was ND, the concentration was replaced with the limit of detection for statistical analysis. Model P values in the three far right columns and post-hoc P values next to the excretion rates are indicated as follow: * P < 0.05; ** P <0.01; *** P<0.001; **** P<0.0001. ND; not detected; CON control. | | | | | | | | | | | | | | | | |
